# Integration of exercise prescription into medical provision as a treatment for non-communicable diseases: A scoping review

**DOI:** 10.3389/fpubh.2023.1126244

**Published:** 2023-07-12

**Authors:** Dan Tao, Roger Awan-Scully, Alistair Cole, Yang Gao, Garrett I. Ash, Yaodong Gu, Frederic Dutheil, Yan Sun, Julien S. Baker

**Affiliations:** ^1^Research Academy of Medicine Combining Sports, Ningbo No. 2 Hospital, Ningbo, China; ^2^Department of Government and International Studies, Hong Kong Baptist University, Kowloon Tong, Hong Kong SAR, China; ^3^Centre for Health and Exercise Science Research, Population Health and Medical Informatics, Hong Kong Baptist University, Kowloon Tong, Hong Kong SAR, China; ^4^Center for Medical Informatics, Yale School of Medicine, Yale University, New Haven, CT, United States; ^5^CNRS, LaPSCo, Physiological and Psychosocial Stress, CHU Clermont-Ferrand, University Hospital of Clermont-Ferrand, Preventive and Occupational Medicine, University Clermont Auvergne, Clermont-Ferrand, France

**Keywords:** non-communicable diseases, exercise prescription, inpatient, outpatient, healthcare service, health policy

## Abstract

**Background:**

The purpose of this scoping review is to stimulate interest and to raise awareness, among researchers, healthcare practitioners, and policymakers regarding the current scientific literature related to exercise prescription for non-communicable diseases (NCDs). Exercise prescription is a safe and cost-effective method that enables physicians to use exercise as a complementary addition to NCDs management and treatment.

**Methods:**

This scoping review followed the PRISMA Extension Guidelines for Scoping Reviews (PRISMA-ScR): Checklist and Explanation. Using this framework, we considered information from qualitative and quantitative studies to identify research gaps. We provide feasible suggestions to guide future research for the implementation of exercise prescription in the healthcare environment. The literature search was conducted using SPIDER and PICO tools for qualitative, quantitative, and mixed-study designs. Inclusion criteria included articles that investigated patients with NCDs and considered exercise interventions. Systematic searches of PubMed, Web of Science, MEDLINE, EMBASE, and ScienceDirect were undertaken on 26 July 2022 and all reference lists were manually searched. Data processing was performed using EndNote 2.0 software and data charts were used for numerical summary and thematic analysis.

**Results:**

There were 10,951 articles retrieved, of which 28 met the inclusion criteria. Based on the evidence, exercise was a feasible, safe, and acceptable method to prevent and manage non-communicable diseases in inpatient and outpatient settings. Six research directions were identified and discussed. In addition, implementation evidence and suggestions for policy-reconfiguration are also provided.

**Conclusion:**

This scoping review summarizes the current evidence for the effectiveness of exercise in the treatment of non-communicable diseases. The review provides key findings supporting exercise prescription for the inpatient and outpatient healthcare service. We suggest that governments and healthcare policymakers globally advocate the inclusion of structured exercise prescription within the NCDs treatment setting.

## 1. Introduction

The Exercise is Medicine (EIM) initiative was launched in 2007 by the American College of Sports Medicine (ASCM). The concept includes a professional physical activity assessment and standardized promotion in clinical care. Exercise is Medicine connects healthcare with valid, evidence-based physical activity and exercise resources for individuals around the world that is inclusive of all health scenarios. The EIM scheme called for key stakeholders and clinicians to provide consensus in support of the development of infrastructures in healthcare provision to provide the inclusion of exercise into routine patient care (1). In spite of this initiative, there has been little change by the medical profession in the prescription of exercise for healthcare provision. Exercise is a validated methodology and applied intervention that helps individuals to develop healthy lifestyles, addresses related illnesses, promotes individuals’ self-esteem, and improves health and wellness (2). There is clear and consistent scientific evidence outlining the benefits of regular exercise interventions on the primary and secondary prevention of diabetes, hypertension, cancers, depression, osteoporosis, and dementia (3). Exercise prescription, physician counseling and referrals for physical activity, can be seen as a non-pharmaceutical treatment during primary and secondary preventions for reducing morbidity and mortality rates from NCDs (4). Many systematic reviews and meta-analyses have demonstrated comprehensive benefits obtained from exercise activity. Exercise prescription can often provide similar or even greater benefits than pharmaceutical interventions, without problematic side effects and associated financial burdens. These observations provide strong evidence for the inclusion of exercise into healthcare provision systems (5–10).

Non-communicable diseases, inclusive of heart disease, stroke, cancer, diabetes and chronic lung disease, etc., are responsible for 74% of deaths globally. This percentage is not equally distributed: 86% of patients dying prematurely or before reaching 70 years are inhabitants of low- and middle-income countries (11). NCDs are also known as chronic diseases, and are predisposed to being of long durations. NCDs normally result from a combination of genetic, physiological, environmental, medical care and behavioral factors (11). Adults, the elderly, and children are vulnerable to the factors contributing to NCDs. These include poor diets, lack of physical activity, and the harmful effects of alcohol and tobacco smoke, etc. (11). However, the most important factors contributing to health outcomes are individual lifestyles and behavior (12).

Globally, it has been suggested that between the years 2011 and 2030, the prevalence of NCDs will result in a cumulative global loss in productivity of US$47 trillion if current trends are not rapidly reversed (13). In poor-resource settings, medication and healthcare costs for the treatment of non-communicable diseases quickly exhaust both household and/or public resources. The costs of NCDs, including treatment, is often lengthy and expensive. These costs in combination with income loss, annually force millions of people into poverty while stifling social and economic development (11, 14). Patient suffering, and family economic and psychological pressures associated with NCDs, come with profound negative consequences for families, governments (locally and nationally), and societies generally (15). Therefore, in addition to the advantages of exercise activity for reducing morbidity and mortality, there are significant socio-economic reasons for introducing exercise prescription into patients’ treatment programs.

Non-communicable diseases hinder progress toward the agreed 2030 Agenda for Sustainable Development. The agenda includes specified targets for reducing premature deaths from NCDs by one-third by 2030 (11). There is now an urgent need for healthcare systems globally to create the necessary infrastructure and environment to ensure that supervised exercise can be, and is, prescribed as medicine. If the available evidence indicates that exercise is effective as a treatment for NCDs, why has exercise not been recognized globally as a prophylactic, stimulating policy change relating to the healthcare and wellness agenda? For example, the Royal National Orthopedic Hospital (RNOH) NHS Trust in London has provided a working example by becoming one of the first NHS trusts in the United Kingdom to open an “exercise prescription clinic.” The clinic provides counseling for patients on the core tenets of health. These include nutrition, sleep, posture, physical activity, and emotional and mental wellbeing (16). Unfortunately, most physicians, who are traditionally medically trained to manage NCDs, have not been comprehensively trained in exercise prescription at associated medical schools or healthcare institutions (4). Meanwhile, most of the intervention studies designed that use physical activity, exercise interventions, and sports physical therapy, are terminated at the efficacy trial stage, without transfer into best practices for healthcare provision and public health policy-making processes.

It has been suggested by researchers that exercise provides an important therapeutic role in preparing patients for treatment and surgery. Exercise is also important in the management of treatment-related side effects, helping patients recover, and in improving treatment tolerability. Emerging evidence also indicates that there is a potential role for exercise to enhance the effectiveness of other treatments (1). Therefore, it seems critical to examine methods to translate these developments into medical practice ensuring that patients receive optimal care. To our knowledge, there are no current scoping reviews that have evaluated exercise interventions for non-communicable disease treatment in inpatient and outpatient treatment settings. This review was designed to address this deficiency in the existing literature. The two objectives of the review were to: (1) to outline current research on applied exercise prescription for NCDs treatment in inpatient and outpatient settings and (2) to identify potential investigative research areas and discuss exercise implementation to guide future research on NCDs.

## 2. Methodology

### 2.1. Protocol and registration

This scoping review followed guidelines obtained from the Joanna Briggs Methods Manual for Scoping Reviews (17, 18). The review followed the PRISMA Extension for Scoping Reviews (PRISMA-ScR): Checklist and Explanation (19). The protocol was developed and registered on the Open Science Framework at https://osf.io/x25zc/ on 17 August 2022 prior to commencing this review.

### 2.2. Eligibility criteria

The literature search was conducted using both SPIDER (Sample, Phenomenon of Interest, Evaluation, Research type) and PICO (Population, Intervention, Comparisons, Outcome) tools for qualitative, quantitative, and mix-study designs to comply with our research design (20–22). The inclusion criteria included articles with patients who were diagnosed with non-communicable diseases and investigated exercise interventions; and contained information pertinent to our research question. The main types of NCDs considered were cardiovascular diseases, cancers, chronic respiratory diseases, and diabetes. Cardiovascular diseases are responsible for most NCDs deaths and account for 17.9 million annually, followed by cancers (9.3 million), respiratory diseases (4.1 million), and diabetes (1.5 million) (11). Therefore, this study focused on NCDs that included cardiovascular diseases (coronary heart disease, cerebrovascular disease, rheumatic heart disease, stroke, hypertension) (23); cancers (all types of cancers); respiratory diseases (chronic obstructive pulmonary disease (COPD), asthma, occupational lung diseases and pulmonary hypertension) (24); and diabetes (type-1 diabetes, type-2 diabetes, and gestational diabetes) (25).

In line with the research aims, studies were included if they met the following criteria: (1) participants/patients diagnosed with non-communicable diseases; (2) studies only evaluating traditional body-type (upper and lower body inclusive of aerobic and anaerobic) exercise interventions (except the studies that only evaluated breathing exercises and oral exercise); (3) the exercise interventions had to provide a well-defined structure was located in an inpatient or outpatient setting and was followed specifically by the patients; (4) studies had to measure and discuss outcomes that related to the research aims; (5) studies were published in English and in peer-reviewed journals within the last decade. All of the following types of studies were excluded: (1) non-primary/original research, including secondary analysis, reviews, commentaries, opinion articles, and viewpoint articles were further excluded to avoid duplication of results; (2) studies where authors could not be contacted to retrieve full texts; (3) studies with data that were not reliably extracted, and animal experiments. If more than one article described a single study presenting the same data, we included the most recent. No exclusion criteria related to age and sex of the patients, medical status, or to culture/sub-cultural factors, geographic location, or race were applied.

### 2.3. Search strategy

An initial limited search of PubMed was performed to identify relevant articles, ensuring the validity of the proposed idea, confirming and prescribing search items, avoiding duplication of previously addressed questions, and assuring that there were enough articles for conducting the analysis. In the preliminary search, no current scoping reviews focusing on exercise interventions with non-communicable diseases in inpatient and outpatient settings were noted. Following the preliminary search, a comprehensive systematic search was conducted independently of five electronic databases on 26 July 2022: PubMed, Web of Science, MEDLINE, EMBASE, and ScienceDirect were explored by MeSH terms in the titles and abstracts to identify and review all relevant literature published within a decade. We used the MeSH terms (‘patient*’) AND (‘exercise prescription’ OR ‘exercise intervention*’ OR ‘exercise treatment’ OR ‘exercise activity’ OR ‘physical activity’ OR ‘exercise training’ OR ‘exercise therapy’ OR ‘exercise movement’) AND (‘non-communicable diseases’ OR ‘NCD*’ OR ‘cardiovascular diseases’ OR ‘coronary heart disease’ OR ‘cerebrovascular disease’ OR ‘rheumatic heart disease’ OR ‘heart attack*’ OR ‘stroke’ OR ‘cancer’ OR ‘tumor’ OR ‘oncology’ OR ‘respiratory diseases’ OR ‘chronic obstructive pulmonary disease’ OR ‘asthma’ OR ‘occupational lung diseases’ OR ‘pulmonary hypertension’ OR ‘type 1 diabetes’ OR ‘type 2 diabetes’ OR ‘gestational diabetes’) to search the studies published. Additionally, manual screening for the reference lists of the retrieved and related articles was conducted.

### 2.4. Data charting process

Identified records were exported to EndNote 20 software for de-duplication. The titles and abstracts were screened independently of all retrieved articles, and the potentially relevant full texts of the remaining articles were screened using eligibility criteria. The following data items were extracted from the included studies: the name of the first author, years and region of publication, characteristics of the patients (sample size, sex, age, medical status, etc.), study design, exercise interventions contents, comparison condition, outcome measures, and the implementation findings of each study. Information relating to publication year, gender characteristics sample size, research design, exercise interventions type, non-communicable diseases categories, and implementation of outcomes was numerically summarized. Additionally, a thematic data analysis from the key findings was also performed. The thematic analysis considered: exercise guidelines, recruitment and adherence, acceptability and resources, existing research, study limitation, experimental context, exercise interventions provider, and future research direction. Data extraction was performed using EndNote 2.0 software. Manually created data charts were used for numerical summary and thematic analysis. The first author developed the data extraction form and a second author checked the form. Any disagreements between authors were resolved through discussion and consultation with a third author.

### 2.5. Critical appraisal of individual sources of evidence

Our study aim was to map the evidence related to our research target; therefore, we included all studies that met the inclusion criteria. This method was consistent with standard scoping review methodologies (17, 26). However, we still provided the critical appraisal results. The methodological quality of the studies was assessed by The Joanna Briggs Institute Critical Appraisal Checklist. Four types of checklist were used to assess 20 selected studies (eight studies were not suitable using The Joanna Briggs Institute Critical Appraisal Checklist system due to non-specific research designs) (27). The checklist answers included: Yes, No, Unclear, or Not/Applicable-NA. We assessed the methodological quality of 20 studies and studies only with minor methodological flaws. The individual included studies were assessed independently and any different opinions were resolved through discussion with the third co-author. The individual critical appraisal results are outlined in Supplementary Table S1.^1^

## 3. Results

### 3.1. Selection of sources

**Figure 1:**
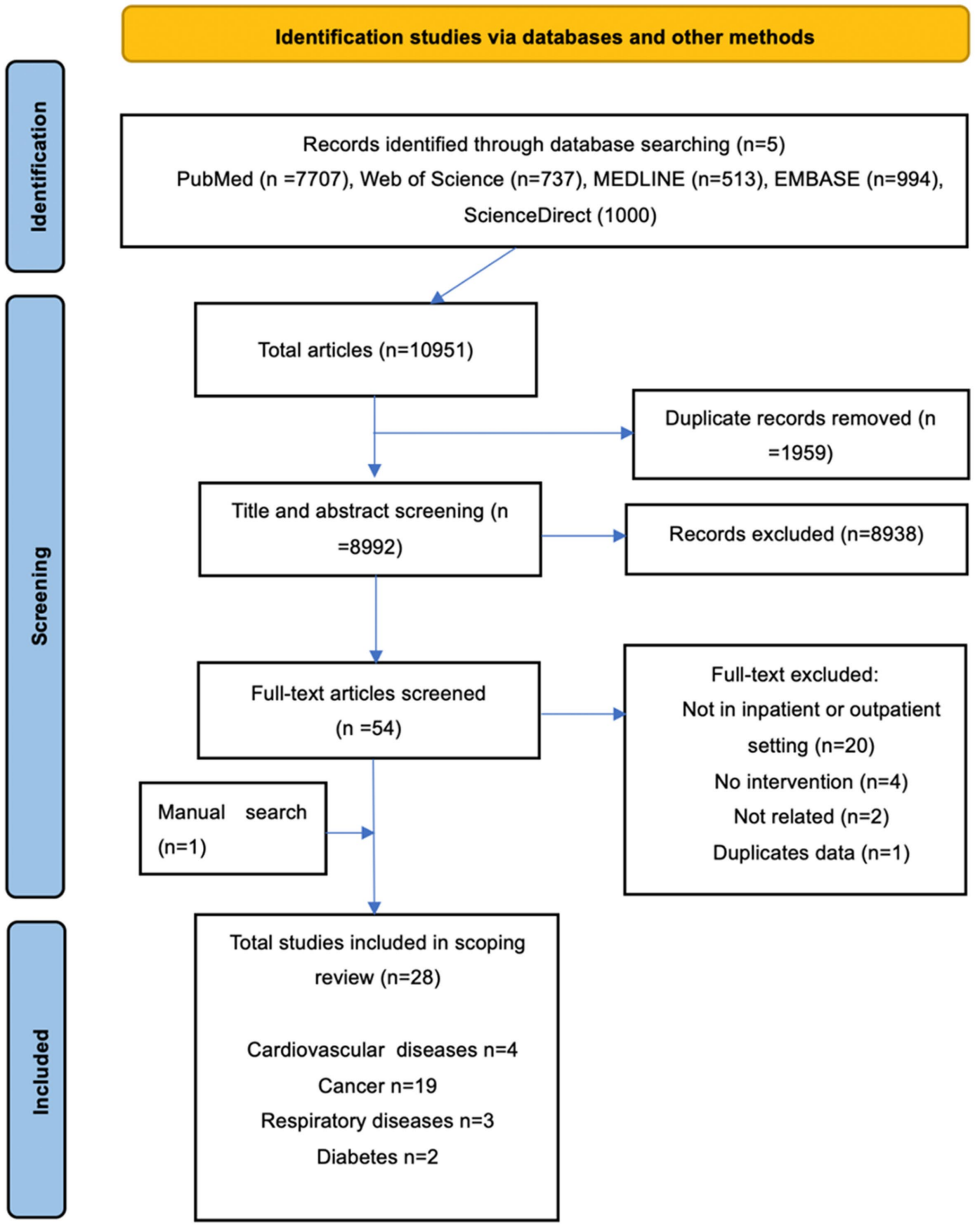
Flowchart of screening and selection process.

From the literature search using five electronic databases, a total of 10,951 articles were retrieved. After the removal of duplicates, title and abstract screening, full-text reading, and manual searching, 28 articles were included in this scoping review. The process of article selection and reasons for exclusion are outlined in Figure 1.

### 3.2. Characteristics of studies

A total of 28 studies comprising 1,368 patients were included in this review. Supplementary Table S2^2^ and Supplementary Table S3^3^ summarize the characteristics of the included studies. Four categories of non-communicable diseases included:

- Studies that focused on cardiovascular diseases (14%): pulmonary arterial hypertension (PAH) (28) and stroke (29–31).
- Cancer-related studies (68%): lung (32–38), colon (39), acute myeloid leukemia and solid tumors (40), breast (37, 41), pediatric cancer (42–46), prostate cancer (PCa) (47), colorectal (48), lower and upper gastrointestinal tract gastrointestinal tract (GI) (37), gynecological (37), multiple myeloma (37), lymphoma (37), leukemia (37, 49), renal (37), other (37), metastatic cancer (50), pancreatic (38), and biliary tract (38).
- Studies for respiratory diseases (11%): chronic obstructive pulmonary disease (COPD) (51–53).
- Diabetes studies (7%): Type 2 diabetes (54, 55).

A total of 21 studies were conducted in the inpatient setting (29–31, 33, 35, 38–42, 44–49, 51–55) and two studies were conducted in an outpatient setting (28, 32). For the remainder of the studies, two were conducted in a mixed-setting (inpatient and outpatient) (34, 43), and three combined hospital-based and home-based exercise interventions (36, 37, 50). Nine of the selected studies were published during 2012–2016 (28, 32–34, 39–41, 51, 54). However, there has been a rapid increase in research studies over recent years, with 19 further studies published during 2016–2022 (29–31, 35–38, 42–50, 52, 53, 55).

A randomized control trial (RCT) research design comprised 54% of the studies (28–33, 35, 36, 39–42, 44, 51, 52); pilot study research designs were used in another 14% (34, 43, 48, 50, 55); the other designs were interviews (10%) (38, 47, 49); experimental designs (10%) (37, 46, 53); a crossover control study (4%) (54); a cohort study (4%) (45); and a feasibility study (4%) (48). In total, 10 types of exercise interventions were used: these included aerobic exercise (28, 32–35, 37–42, 44–47, 49, 50, 52–54); resistance exercise (31, 33–35, 37–42, 44–51, 55); balance exercise (38, 39); core exercise (39); computer-based exercise intervention (43); sling exercise therapy (29); video-guided exercise (30); flexibility exercise (38); respiratory exercise (35); and progressive relaxation exercises (36). The studies all included positive results, except for one. This study obtained negative results demonstrating no significant differences between hospital plus home exercise program and usual care group patients. The findings of the study outlined that all patients had recovered pre-operative exercise tolerance level by 4 weeks after surgery. However, the study also suggested that a post-discharge walking home, following patient evaluation, might provide additional medical benefit for patients (33).

### 3.3. Exercise guidelines

Only eight studies used existing guidelines or were tailored according to previous guidelines, which included a sling exercise therapy (SET) program (29); video-guided exercise (30); exercise program protocol were modeled from existing published cancer exercise and UK physical activity recommendations (47); active video game (Microsoft® Xbox 360 Kinect) (43); enhanced recovery after surgery protocols (ERAS) (48); rehabilitation program in chronic obstructive pulmonary disease (COPD) (35); progressive relaxation exercises protocol (36); and an exercise protocol tailored according to previous studies (45); rest of studies were set using initial exercise prescriptions. More than half of the studies provided detailed exercise protocols for replication for future implementation (30, 31, 33, 34, 36, 37, 39–42, 44, 45, 47, 48, 52). There were several other guidelines contributing to the experimental process. These included the American Thoracic Society 2002 guidelines (35); European Respiratory Society recommendations (35); and Guidelines of the Japan Diabetes Society (55) (see Supplementary Table S4^4^).

### 3.4. Detailed information on exercise intervention

In the selected studies, one study evaluated the acute effects of two types of one-hour exercise interventions for type 2 diabetes patients (54). In other studies, the exercise intervention durations ranged from 2 weeks to 27 weeks, respectively. Some studies’ exercise intervention durations depended on the length of the patients’ hospital stays (39, 49, 51, 52). The length of each individual exercise intervention also varied substantially: in one study being more than 2 h (35); in others 60 ~ 70-min (37, 41, 42, 44–47, 54); in some 30 ~ 45-min (28, 29, 32, 33, 36, 39, 40, 43, 48, 50, 53, 55); or along with conventional therapy (40-min) for an additional 15-min (31), or 15 ~ 45-min (49). The intervention frequency ranged from every day (33, 36, 39, 48, 53, 55); to five times a week (29, 31, 34, 35, 40); three times a week (32, 34, 42, 44, 46); twice a week (28, 38, 41, 45, 47, 49, 50); or once per week (37, 47).

For patients with non-communicable diseases, exercise interventions should be supervised and adjusted by the clinician or physical therapist based on the individual’s exercise response and the medical condition of patients. The studies were conducted using supervised exercise interventions by physical therapists (29, 30, 32, 37, 39, 41, 47, 50, 51); by fitness instructors (42); sport scientists (34, 43, 46, 49); team kinesiologists (48); medical staff (35, 38, 45, 55); or by study investigators (36). Patients in one study expressed that they felt less motivated when exercising independently and most of the participants seemed to prefer a structured, class exercise model as outlined previously (47). Exercise interventions under supervision and team-based not only provide a professional approach and keep the patients safe; in addition, the social environment provides patients with structured classes, and timetabled appointments provide an antidote to motivational inertia.

Exercise intensities were controlled during the studies using several methods. These included heart rate (28, 33, 41, 44, 45, 47); Borg scale (34); VO_2peak_ (54); low-to-moderate exercise intensity (39, 55); (34, 52); based on the patients’ individual health situation (43, 44, 48); based on the baseline measurement results (28, 40, 51); or adjustments by the physical therapist based on individual patients’ response (32). Some exercise programs were adjusted by the physical therapist every 1-2 weeks, every 4 weeks, or increased gradually based on the individual’s response (32, 41, 44, 45, 52). In a study by Platschek, computer-based exercise provided different types and intensities of games based on the patient’s age, individual aerobic capacity, and daily condition (43) (see Supplementary Table S2; see footnote 2).

### 3.5. Acceptability of exercise intervention

The exercise intervention programs used were easily incorporated into an existing clinical rehabilitation program (28). Even 5-year-old patients could actively participate in the workout sessions (46). An important finding was that the studies did not observe any exercise-related adverse events in the selected studies, and all studies agreed that the exercise modalities were feasible and safe for patients. After 30 days following hospital discharge, only one case of wound infection was reported, and none of the patients had to undergo a second surgery and none were readmitted to the hospital during the follow-up period (39). Through the interactions with study and patient education/consulting, patients in the exercise group learned to cope better with their symptoms (32). Motivational force was attributed to peer support, and the friendly environment in the exercise group that provided benefits for the patients’ social development (38, 42, 47). There was an increase in the patients’ interest in exercise activity (43). Furthermore, participants had high confidence to continue exercising following cessation of the program, according to a self-efficacy item provided in one survey (37). Participants described physical and psychosocial benefits from the exercise and commented on the highly valued staff (37). Moreover, participants reported they were satisfied with the program and would recommend it to others (37). Some exercise interventions were easier to conduct: for example, resistance training does not require large spaces in wards/hospital (48) (see Supplementary Table S4; see footnote 4).

### 3.6. Adherence of exercise programs

Patients were recruited from clinics and hospitals in the selected studies. The adherence rate in the four cardiovascular diseases category was 100% in two studies (29, 31); in the other two studies 86% (28) and 79% (30) respectively. With regard to the cancer category (total 19 studies), four studies experienced 100% adherence (42, 43, 45, 46); seven studies were between 80 and 100% (36–41, 48); six studies were between 60 and 80% (32–35, 49, 50); and two studies saw adherence below 60% (44, 47). There were three respiratory disease studies included in this scoping review and the adherence rates were 63% (51), 78% (53), and 100% (52), respectively. Lastly, there were two diabetes studies with adherence rates of 100% (54) (see Supplementary Table S4; see footnote 4).

### 3.7. Resources of exercise implementation

Measurement questionnaires and guidelines that were used in the exercise program can be used as references for future studies and implementation. These included: the Fatigue Severity Scale (FSS) (28), Human Activity Profile (HAP) (28), Motor Status Scale (MSS) (30), General self-efficacy scale (GSE) (30), Trunk impairment Scale (TIS) (31), Stroke-specific quality of life scale (SS-QOL) (31), Borg Rating of Perceived Exertion (RPE) (32–34, 40, 41, 47, 52), Borg CR10 Breathlessness Scale (BBS) (33), Modified Fatigue Impact Scale (MFIS) (40), Hospital Anxiety and Depression Scale (41), PedsQL™ Multidimensional Fatigue Scale (43), Baseline Borg dyspnea scale (35), Dyspnea Index (BDI) (35), Functional Assessment Cancer Therapy-General (FACT-G) (50), PedsQL-4.0 Generic Core Scales (Italian edition) (46), PedsQL Multidimensional Fatigue Scale (Italian edition) (46), Barthel Index (52), Modified Medical Research Council (MMRC) Dyspnea Scale (53), and Cardio-ankle vascular index (55). Questionnaires used were the European Organization for Research and Treatment of Cancer Quality of Life Questionnaire Core 30 (EORTC-QLQ-C30) (40, 50), Short Questionnaire to Assess Health enhancing physical activity (SQUASH) (41), Multidimensional Fatigue Inventory (MFI) (41), Fatigue Quality List (FQL) (41), 30-item European Organization for Research and Treatment of Cancer Quality of Life Questionnaire C30 (41), 36-item Short Form Health Survey (SF-36) (41), Modified paper-and-pencil MoodMeter® questionnaire (43), MILES questionnaire (48), Modified Medical Research Council (mMRC) questionnaire (35), Functional Assessment of Chronic Illness Therapy-Fatigue Questionnaire (FACIT-Fatigue)(50), St. George’s Respiratory Questionnaire (52), Modified Baecke Physical Activity Questionnaire (52), and the St. George’s Respiratory Questionnaire (SGRQ) (53).

The equipment used in the exercise programs included HR monitor (54, 55), Sling suspension equipment (29), Laboratory test equipment (45), Hospital gymnasium (42, 44–46), Cross trainer (47), Metabolic cart (44), Cycle ergometer (34, 40, 47), Resistance bands (48), Weight training machines specifically designed for children (45), Pedometers (33, 38, 53), Dumbbells (47), Portable handheld dynamometer (52), Spirometry (52), Pulse oximeter (52), Treadmill (34, 40, 47, 50), Arm cycling (47), Multigym (47), Active video game (Microsoft® Xbox 360 Kinect) (43), CT (50), In-house software (50), MP3 and earphones (36), Short message service (36), Actiheart; CamNtech—a triaxial accelerometer (54), CGM; Guardian Real-Time with Enlite glucose sensor; Medtronic (54), HBR-2070 (for blood pressure and heart rate measurement) (55), *VS*-1500 (for ankle-brachial index and cardio-ankle vascular index) (55), Mobile tablet (30), and Act iWatch (33) (see Supplementary Table S4; see footnote 4).

### 3.8. Exercise programs providers

Eight studies included exercise sport science specialist, experienced fitness instructor, or kinesiologist (30, 34, 41, 43, 45, 46, 48, 49). In the rest of the studies, physiotherapist (29, 30, 32, 35, 37, 39, 41, 47, 50–52), researcher/investigator (30–33, 35, 36, 39, 41, 42, 44, 45, 47, 49, 51–53), physician/surgeon (34, 35, 39, 44, 48), nurse (36, 38, 39, 48, 53), and hospital/medical staff (36, 37, 39, 45, 55) were involved in the exercise program. No information about exercise prescription providers qualification and years of experience was provided. During the screening of the studies included in this review, it was noticeable that none included physiologists or clinical physiologists in the intervention process to prescribe exercise which would be desirable (see Supplementary Table S4; see footnote 4).

### 3.9. Existing research and limitations

We included four categories of studies for NCDs. For cardiovascular diseases, studies focused on investigating the effectiveness of an exercise intervention for pulmonary arterial hypertension (PAH) (28); and to explore the effectiveness of exercise therapy in stroke patients (29–31). Four studies had the limitations of small sample size (28–31); no sample size calculation (30); or single sex participants (28). Some studies experienced patients withdrawing due to changes in medication (28), or low attendance at the exercise sessions (28). Some results were based on self-reports (28), or had potential performance and social desirability biases (30).

Regarding the cancer category, one study focused on determining the effect of progressive relaxation exercise in patients with lung cancer (36); some studies specially focused on non-small cell lung cancer (NSCLC) (32–35). Two studies examined the effects of a postsurgical, inpatient exercise program in colon cancer patients (39), and investigated the feasibility of initiating resistance exercise in colorectal cancer patients (48). Several studies explored perceived exercise benefits and barriers in adults with acute leukemia (49); patients’ experience of a structured exercise intervention for men with prostate cancer (PCa) (47); and the effects of an exercise intervention on preventing an increase in fatigue in patients with breast cancer (41). For the pediatric cancer area, studies examined the effects of an in-hospital exercise intervention (42, 44–46) and a computer-based exercise intervention in pediatric cancer patients (43). The rest of the four studies examined the feasibility of embedding a flexible, exercise-based rehabilitation program into cancer treatment (37, 50); the effects of aerobic and strength exercise in hospitalized cancer patients (40); and the experiences of older patients with advanced cancer who participated in an exercise program (38), respectively.

Meanwhile, small sample sizes (32, 34, 37, 39, 40, 43, 44, 46, 49, 50) also existed in the cancer studies. Further limitations were difficulties in recruiting participants (32, 44); adherence problems and low attendance for exercise intervention (32–36, 39, 40, 44, 45, 47, 50); heterogeneity in participants’ characteristics (44–46); or non-randomized sampling (37, 45, 46). Considering study design, some exercise studies were unblinded (33, 39, 47), or lacked a control group (34, 43, 47, 48, 50). Measurement limitations included the timing and nature of surgery, meaning that authors were unable to collect preoperative activity data (33); lack of assessment of psychosocial parameters (40); the authors being unable to supervise some processes (33, 37, 50); outcomes being assessed more descriptively instead of being objectively measured and analytical (36, 38, 41, 43, 46, 47, 49); or lack of biochemical marker measurements (35) were also observed weaknesses in some studies. For the outcome results, patients’ familiarization with the testing equipment leading to neural adaptation contributing to increases in performance (42). Other limitations included changing hospital policies (making it impossible to meet the recruitment criteria) (39), patients’ motivations (32), or changes in some of the testing items for patients ‘medical reasons (42).

As for the three respiratory diseases studies, the focus was on measuring the effect of whole-body resistance training in patients hospitalized for exacerbation of chronic obstructive pulmonary disease (COPD) (51); determining the effects of regular walking programs in patients with stage I and II COPD (53); and determining whether an exercise intervention can reduce disability in frail older patients with acute exacerbation of chronic obstructive pulmonary disease (AECOPD) (52). Once again, there were several limitations in the studies reviewed. The test results were influenced by variable patient motivations (51, 52); small sample size (51); adherence problems (51, 53); the patients’ early discharge and low attendance rate for exercise interventions (51); the lack of a biopsy to assess muscle condition (52); or self-reported measurements (53).

The two diabetes studies focused on determining whether interval-based exercise improves postprandial glucose tolerance and free-living glycemia more than oxygen consumption- and time duration-matched continuous exercise(54); and investigating the effect of short-term toe resistance training on toe pinch force and toe muscle quality (55), respectively. The limitations were small sample size (54, 55); research design limitation(54); or the lack of a comparison group (55) (see Supplementary Table S5^5^).

## 4. Discussion

In this scoping review, we selected 28 studies that used exercise interventions with non-communicable diseases patients in inpatient or outpatient settings. There were positive outcomes in 27 studies. These included increasing the patients’ physical activity levels (28, 41); improved cardiorespiratory fitness (28, 53); decreased fatigue (28, 40, 41, 43, 46); improved quality of life (29, 31, 34, 46, 50, 53); relief of pain (29); improved body mobility and capacity (31, 32, 34, 35, 38–42, 44, 50–53, 55); alleviating related symptoms (32, 36, 38, 40, 53); reduced hospital stays (39, 45); and better glycemic control (54). Intervening with exercise-based rehabilitation within the treatment unit represents an opportunity for early support, including behavior change strategies during treatment to prevent deterioration in health status (37). Exercise prescription should be individually prescribed and interventions should be based on clinical examination results and the stage of the disease. In this review, exercise interventions conducted at the same time as chemotherapy (35, 36, 40, 42, 44, 45, 49, 50), or after surgery (33, 39, 48), were found to be feasible and acceptable. Additionally, there were positive effects on psychological aspects in patients involved in exercise (38, 43, 46, 49). Data syntheses of results demonstrated that exercise intervention is a safe, effective, and feasible method for non-communicable diseases’ patients, with no exercise-related adverse events being reported.

The exercise intervention guidelines summarized in this study, indicating that more than half of the included studies offered very detailed exercise intervention plans. The intervention plans were reliable and provide validated evidence for designing exercise programs that can be replicated in future exercise implementations. Furthermore, there are many sophisticated scales and questionnaires that can be used by physicians in the process of exercise interventions for non-communicable diseases treatment. The exercise equipment used was simple to operate and easily accessible for the implementation process. All the studies were conducted in the hospital (three studies combined hospital and home-based exercise intervention), eight of the 28 studies exercise programs were proscribed by exercise specialist, other involved exercise intervention providers such as physiotherapist, nurse, medical staff, physician, investigator, or researcher who provided exercise supervision and support services during the treatment process. Consequently, the exercise guidelines and resources provide the foundation for exercise interventions in the healthcare system.

Functional clinical testing is recommended before prescribing exercise for patients. As part of the pre-participation health screening process, it is recommended that all moderately to high-risk patients undergo a medical examination and/or stress test, and a formal clinical consultation before commencing on an exercise program (56). In our scoping review, more than 80% of the selected studies provided detailed participant inclusion and exclusion criterion. These are useful references providing information for physicians and practitioners to implement exercise prescription and operate an exercise intervention in the non-communicable diseases’ treatment setting. However, adherence rates were a problem in many of the selected studies. The main reasons were due to changes in medication, patients’ motivation for exercise, and the patients’ early discharge from hospital. In addition, patients’ anxiety, aches, and pains cannot be ignored (49). In our review, only two studies mentioned financial issues [Morales’s study reported the economic cost of hospitalization, the significantly lower number of hospitalization days in the exercise group, representing a ~ 17% reduction in economic costs compared with the control group (45). In Dennett’s study, no additional expenses were incurred for equipment or venue. The primary resource cost was the funding of the staff resource (37)].

In a previous study outlining the benefits of exercise for NCDs, Ezenwankwo discussed embedding exercise service units into clinical oncology settings. The research outlined six studies providing data from 30 exercise programs. Issues relating to funding, lack of a detailed implementation plans, and low organizational buy-in were the major barriers to effective service integration, particularly at the health service level (57). A further study by Kennedy, investigating the implementation of exercise into routine cancer care, was hindered by a web of interrelated challenges across all levels of the healthcare system. These challenges limited the ability of patients to access effective exercise resources during cancer treatment (1).

The studies examined here provide strong and consistent evidence that exercise interventions in the NCDs treatment setting, especially in the inpatient situation, can help enhance conventional treatment methods. Exercise prescription contains both effective and enjoyable activities to match individual patients’ preferences, ability, and limitations. Exercise interventions supervised by experienced health practitioners (preferably by the same supervisor throughout the whole duration of the programs) can demonstrate complementary advantages during treatment. Also, optimally coordinated with medical appointments, a holistic, multidisciplinary approach that includes symptom monitoring, provision of advice on symptom management, systematic assessment of patients’ health status and information on individual life situations, will increase treatment compatibility. Patients who receive professional guidance from their physician or physiological expertise in the health care team in inpatient and outpatient treatment consultations, will likely develop greater confidence related to the positive effects of exercise and continue exercise routines after discharge.

### 4.1. Suggestions for implementation

Exercise interventions for NCDs patients are feasible and acceptable for both the medical provider and patient. The evidence indicates that exercise is complementary to conventional therapy, with no adverse events. Detailed information relating to exercise guidelines, and the medical resources in the hospital environment from 28 studies, provide reliable evidence for future practice and implementation.

To bridge the gap from theory to practical implication and the realization of aims, in addition to the scientific policy-making process and suggestions, consideration needs to be given to a general alignment between policy and practice to ensure long-term and effective implementation and delivery. These processes are normally completed by policy actors who are individuals or groups that are directly or indirectly, formally or informally, affiliated with or affected by the policy process at any stage. The roles of the actors in this case include several related groups: the hospital where exercise prescription is especially used; medical school, universities that provide resources for physicians to become qualified to prescribe exercise; medical insurers in some care systems; and even the business organizations that provide the resources for exercise tests and exercise practice equipment. The functions of the actors are to promote patients’ healthy behaviors; building and developing capacity such as physician’s exercise prescription skills and physiotherapist numbers; improving access to healthcare and other essential goods and health service; changing the hospital and social healthcare institutions attitudes for exercise as a medicine; redistribution of financial or other medical resources, etc. The context for exercise prescription delivery comprises the hospital setting, equipment, medical training system, the physician’s skill and ability to provide exercise prescription, and the patients’ confidence in the benefits of exercise prescription.

The implementation of exercise prescription will require the coordination by multiple stakeholders including government agencies, politicians, non-government organizations, professional societies, legislatures, healthcare systems, and the healthcare industry (58). Additionally, medical schools, hospital services, and patients exercise education need mobilization for exercise prescription to gain momentum as a formal treatment for non-communicable diseases. The best scenario would include a governmental shift in health provision that includes collaboration with exercise professionals in inpatient and outpatient healthcare provision, along with a re-orientation of the general practice environment toward an exercise health promotion initiative. At the societal level, healthcare support includes developing a practical infrastructure, equipment base, peer networks, and improvements in the confidence of patients for exercise intervention, making them more receptive.

### 4.2. Future directions

Several suggestions for future research emanating from this scoping review include the following:

- In this study, most of the articles included were retrieved from cancer studies. There are also many original research articles, systematic reviews and meta-analyses conducted investigating cardiovascular diseases (59–62), respiratory diseases (63–65), and diabetes (66–69); however, studies pertaining to exercise prescription in the inpatient and outpatient environment for these three types of non-communicable diseases are limited. Based on this, more RCT studies are needed in the hospital environment to explore the effects of exercise interventions. Further research needs to include larger sample sizes; to explore the optimal duration, intensity, and frequency of exercise training; and further experimentation investigating the design, durability, and generalizability of exercise programs. Additionally, following clinical assessment and to measure the postoperative complications after discharge, wearable device initiatives need to be considered and implemented.
- There is a need for (combined qualitative and quantitative methods) research investigating different ethnic groups, and whether results are consistent across studies. Also, more objective and precise measurements are required in future studies.
- There is also a need to estimate the beneficial effects of exercise interventions in isolation without medication.
- Future work needs further qualitative studies to elucidate both patients’ and clinicians’ attitudes, motivation and confidence toward exercise as a medicine, and explore the barriers toward participation in exercise programs using a multidisciplinary perspective in order to translate evidence into practice and improve patient outcomes.
- There is a need for further studies that focus on financial issues, such as insurance policy implementation, and the cost of exercise prescription etc.
- More research is required to confirm the benefits of exercise intervention in health service provision, to drive policy-changing and the funding exercise prescription as part of standard care.

### 4.3. Strengths and limitations

The major strength of this review is that the information provided demonstrates the consistent and substantial benefits of exercise as medicine for patients with non-communicable diseases. A further strength is that the article outlines adequate existing resources for exercise as medicine, and identifies barriers that need to be overcome for success in implementation of the findings in the health-policy-making process. This study has updated the exercise benefits for treatment methods regarding NCDs, outlined the need for future research and indicated where there were discrepancies in the literature. This study has also provided meaningful suggestions for future research directions.

There were some study limitations. Firstly, there were flaws in the experimental design of some of the selected studies. We did not set any restriction for study design and only restricted the exercise interventions conducted in inpatient or outpatient NCDs treatment settings. Therefore, there were problems with small sample sizes; no sample size calculations; self-reported results; single sex participants; lack of control groups; outcomes being assessed more descriptively instead of using objective measurements; outcomes not being analytical; or non-randomized sampling problems. Secondly, we were unable to provide the exercise cost in the inpatient and outpatient setting. Also, the patients’ satisfaction level for the exercise interventions of selected studies was not provided. Lastly, there were large data sets for cancer studies, but for the other three NCDs investigated there was less data available for inclusion.

## 5. Conclusion

Based on the validated evidence, we conclude that the addition of exercise is a powerful complementary treatment method to prevent and manage non-communicable diseases. Exercise prescription as a non-pharmaceutical health intervention can be promoted and implemented in combination with traditional medical science. This medical management treatment approach may be helpful in providing the basis of a new healthcare service model. This review summarizes the evidence and suggests implementing exercise prescription into non-communicable diseases treatment settings in the inpatient and outpatient environment. This would improve the status of the population’s health and enhance healthy lifestyles globally, while reducing the social and economic costs associated with NCDs.

## Author contributions

DT and AC drafted the study design. RA-S, JB, GA, YDG, FD, and YS provided critical feedback on the protocol. DT and JB did the literature searches. DT and RA-S contributed to the screening process and selection of included studies. DT initially extracted the data and did the qualitative and quantitative data analysis, and it subsequently verified by RA-S. DT completed the data synthesis. All authors had access to the data, critically reviewed and approved the manuscript.

## Conflict of interest

The authors declare that the research was conducted in the absence of any commercial or financial relationships that could be construed as a potential conflict of interest.

## Publisher’s note

All claims expressed in this article are solely those of the authors and do not necessarily represent those of their affiliated organizations, or those of the publisher, the editors and the reviewers. Any product that may be evaluated in this article, or claim that may be made by its manufacturer, is not guaranteed or endorsed by the publisher.

## Footnotes

^1^https://osf.io/mgbst

^2^https://osf.io/b2jkq

^3^https://osf.io/tvp4w

^4^https://osf.io/jwv7y

^5^https://osf.io/b3z4f
