# Supplementary material for: Integration of exercise prescription into medical provision as a treatment for non-communicable diseases: A scoping review
Source: Front Public Health. 2023 Jul 12;11:1126244. doi: 10.3389/fpubh.2023.1126244 (PMC10369190; doi:10.3389/fpubh.2023.1126244)
Supplement: Supplementary file 1 [file Table_1.DOCX]

**Table 1. Critical Appraisal for Including Studies** (The Joanna Briggs Institute checklists)

|  | Citation | Checklist Items |  |  |  |  |  |  |  |  |  |  |  |  |  |
| --- | --- | --- | --- | --- | --- | --- | --- | --- | --- | --- | --- | --- | --- | --- | --- |
| **RCT** |  | Was true randomization used for assignment of participants to treatment groups? | Was allocation to treatment groups concealed? | Were treatment groups similar at the baseline? | Were participants blind to treatment assignment? | Were those delivering treatment blind to treatment assignment? | Were outcomes assessors blind to treatment assignment? | Were treatment groups treated identically other than the intervention of interest? | Was follow up complete and if not, were differences between groups in terms of their follow up adequately described and analyzed? | Were participants analysed in the groups to which they were randomized? | Were outcomes measured in the same way for treatment groups? | Were outcomes measured in a reliable way? | Was appropriate statistical analysis used? | Was the trial design appropriate, and any deviations from the standard RCT design (individual randomization, parallel groups) accounted for in the conduct and analysis of the trial? | Overall appraisal Included; Excluded; Seek further info |
|  | Liu, J.  2020(1) |  |  |  |  |  |  |  |  |  |  |  |  |  |  |
|  | Kenny, M.  2022(2) |  |  |  |  |  |  |  |  |  |  |  |  |  |  |
|  | Mahmood, W.  2022(3) |  |  |  |  |  |  |  |  |  |  |  |  |  |  |
|  | Hwang, C.L.2012(4) |  |  |  |  |  |  |  |  |  |  |  |  |  |  |
|  | Ahn,K.Y.  2013(5) |  |  |  |  |  |  |  |  |  |  |  |  |  |  |
|  | Arbane, G.  2014(6) |  |  |  |  |  |  |  |  |  |  |  |  |  |  |
|  | Travier, N.  2015(7) |  |  |  |  |  |  |  |  |  |  |  |  |  |  |
|  | Morales, J. S.  2018(8) |  |  |  |  |  |  |  |  |  |  |  |  |  |  |
|  | Rutkowska, A.  2019(9) |  |  |  |  |  |  |  |  |  |  |  |  |  |  |
|  | Kirca, K.  2021(10) |  |  |  |  |  |  |  |  |  |  |  |  |  |  |
|  | Borges, R. C.  2014(11) |  |  |  |  |  |  |  |  |  |  |  |  |  |  |
|  | Fiuza-Luces, C.  2017(12) |  |  |  |  |  |  |  |  |  |  |  |  |  |  |
|  | Torres-Sanchez, l.  2017(13) |  |  |  |  |  |  |  |  |  |  |  |  |  |  |
|  | Weinstein, A. A.  2013(14) |  |  |  |  |  |  |  |  |  |  |  |  |  |  |
|  | Oechsle,K.  2014(15) |  |  |  |  |  |  |  |  |  |  |  |  |  |  |
| **Cohort study** |  | Were the two groups similar and recruited from the same population? | Were the exposures measured similarly to assign people to both exposed and unexposed groups? | Was the exposure measured in a valid and reliable way? | Were confounding factors identified? | Were strategies to deal with confounding factors stated? | Were the groups/participants free of the outcome at the start of the study (or at the moment of exposure)? | Were the outcomes measured in a valid and reliable way? | Was the follow up time reported and sufficient to be long enough for outcomes to occur? | Was follow up complete, and if not, were the reasons to loss to follow up described and explored? | Were strategies to address incomplete follow up utilized? | Was appropriate statistical analysis used? |  |  |  |
|  | Morales, J. S.  2020(16) |  |  |  |  |  |  |  |  |  |  |  |  |  |  |
| **Qualitative research** |  | Is there congruity between the stated philosophical perspective and the research methodology? | Is there congruity between the research methodology and the research question or objectives? | Is there congruity between the research methodology and the methods used to collect data? | Is there congruity between the research methodology and the representation and analysis of data? | Is there congruity between the research methodology and the interpretation of results? | Is there a statement locating the researcher culturally or theoretically? | Is the influence of the researcher on the research, and vice- versa, addressed? | Are participants, and their voices, adequately represented? | Is the research ethical according to current criteria or, for recent studies, and is there evidence of ethical approval by an appropriate body? | Do the conclusions drawn in the research report flow from the analysis, or interpretation, of the data? |  |  |  |  |
|  | Fox, L.  2017  London, UK(17) |  |  |  |  |  |  |  |  |  |  |  |  |  |  |
|  | Mikkelsen, M. K.  2022(18) |  |  |  |  |  |  |  |  |  |  |  |  |  |  |
|  | Leak Bryant, A  2017(19) |  |  |  |  |  |  |  |  |  |  |  |  |  |  |
| **Quasi-Experimental** |  | Is it clear in the study what is the ‘cause’ and what is the ‘effect’ (i.e. there is no confusion about which variable comes first)? | Were the participants included in any comparisons similar? | Were the participants included in any comparisons receiving similar treatment/care, other than the exposure or intervention of interest? | Was there a control group? | Were there multiple measurements of the outcome both pre and post the intervention/exposure? | Was follow up complete and if not, were differences between groups in terms of their follow up adequately described and analyzed? | Were the outcomes of participants included in any comparisons measured in the same way? | Were outcomes measured in a reliable way? | Was appropriate statistical analysis used? |  |  |  |  |  |
|  | Spreafico, F.  2021(20) |  |  |  |  |  |  |  |  |  |  |  |  |  |  |

Answers: Yes, No, Unclear or Not/Applicable-NA

**References**

1. Liu J, Feng W, Zhou J, Huang F, Long L, Wang Y, et al. Effects of sling exercise therapy on balance, mobility, activities of daily living, quality of life and shoulder pain in stroke patients: a randomized controlled trial. Eur J Integr Med [Internet]. 2020;35(February):101077. Available from: https://doi.org/10.1016/j.eujim.2020.101077

2. Kenny M, Gilmartin J, Thompson C. Video-guided exercise after stroke: a feasibility randomised controlled trial. Physiother Theory Pract. 2020;1–12.

3. Mahmood W, Ahmed Burq HSI, Ehsan S, Sagheer B, Mahmood T. Effect of core stabilization exercises in addition to conventional therapy in improving trunk mobility, function, ambulation and quality of life in stroke patients: a randomized controlled trial. BMC Sports Sci Med Rehabil. 2022;14(1):1–9.

4. Hwang CL, Yu CJ, Shih JY, Yang PC, Wu YT. Effects of exercise training on exercise capacity in patients with non-small cell lung cancer receiving targeted therapy. Supportive Care in Cancer. 2012;20(12):3169–77.

5. Ahn KY, Hur H, Kim DH, Min J, Jeong DH, Chu SH, et al. The effects of inpatient exercise therapy on the length of hospital stay in stages I-III colon cancer patients: Randomized controlled trial. Int J Colorectal Dis. 2013;28(5):643–51.

6. Arbane G, Douiri A, Hart N, Hopkinson NS, Singh S, Speed C, et al. Effect of postoperative physical training on activity after curative surgery for non-small cell lung cancer: A multicentre randomised controlled trial. Physiotherapy (United Kingdom) [Internet]. 2014;100(2):100–7. Available from: http://dx.doi.org/10.1016/j.physio.2013.12.002

7. Travier N, Velthuis MJ, Steins Bisschop CN, van den Buijs B, Monninkhof EM, Backx F, et al. Effects of an 18-week exercise programme started early during breast cancer treatment: A randomised controlled trial. BMC Med [Internet]. 2015;13(1):1–11. Available from: http://dx.doi.org/10.1186/s12916-015-0362-z

8. Morales JS, Padilla JR, Valenzuela PL, Santana-Sosa E, Rincón-Castanedo C, Santos-Lozano A, et al. Inhospital exercise training in children with cancer: Does it work for all? Front Pediatr. 2018;6(December):1–8.

9. Rutkowska A, Jastrzebski D, Rutkowski S, Zebrowska A, Stanula A, Szczegielniak J, et al. Exercise Training in Patients With Non-Small Cell Lung Cancer During In-Hospital Chemotherapy Treatment: A RANDOMIZED CONTROLLED TRIAL. J Cardiopulm Rehabil Prev. 2019;39(2):127–33.

10. Kırca K, Kutlutürkan S. The effect of progressive relaxation exercises on treatment-related symptoms and self-efficacy in patients with lung cancer receiving chemotherapy. Complement Ther Clin Pract. 2021;45(September).

11. Borges RC, Carvalho CR. Impact of resistance training in chronic obstructive pulmonary disease patients during periods of acute exacerbation. Arch Phys Med Rehabil [Internet]. 2014;95(9):1638–45. Available from: http://dx.doi.org/10.1016/j.apmr.2014.05.007

12. Fiuza-Luces C, Padilla JR, Soares-Miranda L, Santana-Sosa E, Quiroga J v., Santos-Lozano A, et al. Exercise Intervention in Pediatric Patients with Solid Tumors: The Physical Activity in Pediatric Cancer Trial. Med Sci Sports Exerc. 2017;49(2):223–30.

13. Torres-Sánchez I, Valenza MC, Cabrera-Martos I, López-Torres I, Benítez-Feliponi Á, Conde-Valero A. Effects of an Exercise Intervention in Frail Older Patients with Chronic Obstructive Pulmonary Disease Hospitalized due to an Exacerbation: A Randomized Controlled Trial. COPD: Journal of Chronic Obstructive Pulmonary Disease. 2017;14(1):37–42.

14. Weinstein AA, Chin LMK, Keyser RE, Kennedy M, Nathan SD, Woolstenhulme JG, et al. Effect of aerobic exercise training on fatigue and physical activity in patients with pulmonary arterial hypertension. Respir Med [Internet]. 2013;107(5):778–84. Available from: http://dx.doi.org/10.1016/j.rmed.2013.02.006

15. Oechsle K, Aslan Z, Suesse Y, Jensen W, Bokemeyer C, de Wit M. Multimodal exercise training during myeloablative chemotherapy: A prospective randomized pilot trial. Supportive Care in Cancer. 2014;22(1):63–9.

16. Morales JS, Santana-Sosa E, Santos-Lozano A, Baño-Rodrigo A, Valenzuela PL, Rincón-Castanedo C, et al. Inhospital exercise benefits in childhood cancer: A prospective cohort study. Scand J Med Sci Sports. 2020;30(1):126–34.

17. Fox L, Cahill F, Burgess C, Peat N, … SRB research, 2017 undefined. Real world evidence: a quantitative and qualitative glance at participant feedback from a free-response survey investigating experiences of a structured exercise. HindawiCom [Internet]. 2017;2017. Available from: https://www.hindawi.com/journals/bmri/2017/3507124/abs/

18. Mikkelsen MK, Michelsen H, Nielsen DL, Vinther A, Lund CM, Jarden M. ‘Doing What only I Can Do’: Experiences from Participating in a Multimodal Exercise-Based Intervention in Older Patients with Advanced Cancer - A Qualitative Explorative Study. Cancer Nurs. 2022;45(2):E514–23.

19. Bryant AL, Walton AML, Pergolotti M, Phillips B, Bailey C, Mayer DK, et al. Perceived benefts and barriers to exercise for recently treated adults with acute leukemia. Oncol Nurs Forum. 2017;44(4):413–20.

20. Spreafico F, Barretta F, Murelli M, Chisari M, Gattuso G, Terenziani M, et al. Positive Impact of Organized Physical Exercise on Quality of Life and Fatigue in Children and Adolescents With Cancer. Front Pediatr. 2021;9(June):1–10.
