# Supplementary material for: Integration of exercise prescription into medical provision as a treatment for non-communicable diseases: A scoping review
Source: Front Public Health. 2023 Jul 12;11:1126244. doi: 10.3389/fpubh.2023.1126244 (PMC10369190; doi:10.3389/fpubh.2023.1126244)
Supplement: Supplementary file 2 [file Table_2.DOCX]

**Table 2. The Characteristics Summary of the Studies**

| **Author/**  **Publication year/Region** | **Patients Characteristics** | **Study Design** | **Exercise Interventions’ Frequency/Intensity/Duration/ Type** | **Comparison Condition** | **Outcome Measures** | **Implementation Findings** |
| --- | --- | --- | --- | --- | --- | --- |
| 1. Weinstein, A. A.   2013  USA(1) | - Sample size: 28 - Sex: Female - Age: 54.4 ±10.4 years old - Diagnosis of pulmonary arterial hypertension (PAH) at least three months - Inclusion: Not pregnant, tobacco free - No participant in structured aerobic exercise three days a week for 30-min or more - Exclusion: participant in structured aerobic exercise three days a week for 30-min or more; if WHO function class I and could walk more than 400-m or WHO function IV and could not walk more than 50-m during the Endurance-6-min walk test (6MWT); FEV1/FVC ratio ≤ 65%; a history of ischemic heart disease; an ejection fraction < 40% or a documented pulmonary capillary wedge pressure ≥ 18 mmHg; significant hepatic, renal, metabolic or mitochondrial dysfunctions; severe psychiatric disease; use of beta-adrenergic blockers or antiretroviral therapies; and any musculoskeletal or neurological condition | Randomized control trial (RCT) | - Medically supervised treadmill waking - 30~45-min pre-session, two times pre-week for 10-week - Intensity range of 70%~80% of each subject’s heart rate - Education sessions-20 lectures, one hour every lecture, over 10-week | Education plus aerobic exercise training (EXE) vs Education portion of the regimen (EDU) | - Fatigue Severity Scale (FSS) - The Human Activity Profile (HAP) - Endurance-6-min walk test (6MWT) - The symptom limited treadmill exercise test | - Higher levels of physical activity in Education plus aerobic exercise training - Improve cardiorespiratory fitness and Endurance-6-min walk test distance in Education plus aerobic exercise training - Decrease in fatigue severity in Education plus aerobic exercise training |
| 1. Liu, J.   2020  Hengyang, Hunan, China(2) | - Sample size: 50 - Male: 35, Female: 15 - Age: 56.52 ± 9.22 years old in intervention group, 56.6 ± 9.12 years old in control group - Stroke patients - Inclusion: diagnosed as having initial cerebral hemiplegia by magnetic resonance imaging (MRI) or computerized tomography (CT); age 20~70 years old; course of disease within six months, with stable vital signs; no balance disorders before this stroke - Exclusion: serious viscera dysfunction; serious joint diseases; history of mental illness or sever cognitive impairment, audio-visual understanding obstacle, unable to cooperate with instructions; infection and ulcers on skin | Assessor-blinded Randomized control trial (RCT) | - Sling exercise therapy - Once a day for 30-min, five times per-week for 4-week - In addition, all participators received comparable routine rehabilitation treatments other than sports training and individualized drug treatment | Sling exercise therapy group vs Control group | - The Berg Balance Scale - Motor function - Barthel index - Quality of life - Shoulder pain - Pain | - The sling exercise therapy has been shown to be a safe and effective method to improve balance, mobility, activities of daily living, quality of life and shoulder pain in stroke patients - In particular, it was superior to the control group in balance, upper limb motor function and shoulder pain |
| 1. Kenny, M.   2022  UK(3) | - Sample size: 14 (11 completed) - Male: 6, Female: 8 - Age: (Mean) 73.5 years old - Participants were newly diagnosed stroke inpatients - Inclusion: age be 18 or over years old; diagnosed with first time stroke; mild or moderate upper limb paresis; no previous/existing upper limb pathology; not enrolled in another trail; predicted four-week hospital stay; mild/moderate or no aphasia and understood consent | Randomized control trial (RCT) | - Participants had out-of-therapy-time exercises provided by video on a mobile tablet - Trial period was 4-week - No prescribed amount of exercise was provided and participants were free to undertake exercises as often as they wished | Video guide on a tablet to support exercise group vs Control group (treatment as usual) | - Quantitative data   Primary outcome was impairment and disability  Secondary outcomes included quality of movement, self-efficacy, and time, minutes of spent engaged exercises   - Qualitative data   Interview | - A trial of video-guided exercise is feasible, although an optimal main trial would require some relatively minor changes to design, outcome measures, eligibility, and the intervention |
| 1. Mahmood, W.   2022  Pakistan(4) | - Sample size: 41 - Male: 26, Female: 15 - Age: 57.10 ± 6.28 years old in intervention group, 54.95 ± 6.35 years old in control group - Chronic ischemic stroke - Inclusion: first-time stroke, more than six months, not more than one year; age 45~65 years old; who have achieved sitting for at least 10s and had a definite diagnosis of stroke confirmed through magnetic resonance imaging (MRI) or computerized tomography (CT) - Exclusion: severe cognitive and communication disorders; any neurological and sensory disorders other than stroke; any visual, sensory, and hearing impairment that was not corrected through aids; any metabolic and malignant disorder | Assessor Blinded Randomized control trial (RCT) | - Core stability training along with conventional (40-min) therapy for additional 15-min by increasing the frequency of repetition from 10 to 20 repetitions according to patient condition - Core stability consisted of abdominal drawing-in maneuver (ADIM) for contraction of transverses abdominis - Each patient received five treatment sessions per-week for 8-week | Exercise intervention vs Control group | Main measures:   - Trunk impairment scale (TIS) - Functional ambulation category (FAC) - Stroke specific quality of life (SSQOL) - Trunk range of motion (ROM) | - This study concluded that core stabilization training was better as compared to the conventional physical therapy treatment for improving trunk impairments, functional ambulation and quality of life among patients of stroke - The core stabilization training was also more effective in improving trunk mobility in sagittal plane |
| 1. Hwang, C.L.   2012  Taiwan(5) | - Sample size: 24 - Male: 12, Female:12 - Age: 61.0 ± 6.3 years old in intervention group, 58.5 ± 8.2 years old in control group - Participants: diagnosis of adenocarcinoma for more than four weeks and an Eastern Cooperative Oncology Group performance status of 0 or 1; the patients at advanced stages who were receiving targeted therapy - Inclusion: medically stable and only received epidermal growth factor receptor inhibitors for ≥ 4 weeks; - Exclusion: diagnosis of diabetes; an unstable condition from metastasis; primary lung disease other than lung cancer; sever cardiac or musculoskeletal conditions; inability to understand verbal or written instructions | Randomized control trial (RCT) | - Treadmill or cycling ergometer - three times a week for 24 sessions, each exercise session was 30~40-min - The exercise program including interval intensity and duration was adjusted by the physical therapist every 1~2-week based on the individual’s exercise response | Exercise group vs Control group  Control group received the usual care, general patient education, and social phone calls every 2-3 weeks without supervised exercise intervention | - Assessments of VO_2peak_, muscle strength, endurance and oxygenation during exercise - Insulin resistance, inflammatory response - Quality of life   All tests were performed by a blinded assessor | - No exercise-related adverse events were reported - Exercise training appears to improve exercise capacity and alleviate some cancer-related symptoms - No effect of muscle strength, endurance, insulin resistance, or inflammatory response |
| 1. Ahn,K.Y.   2013  Seoul, Korea(6) | - Sample size: 36 - Male: 17, Female: 14 (completed) - Age: 55.61 ± 7.11 years old in intervention group, 57.43 ± 6.12 years old in control group - Participant: stage I to III colon cancer - Inclusion: postoperative; ability to read and understand Korean; willingness to participate in the study even after understanding the randomization process - Exclusion: recurrent or metastatic disease; immunosuppressant medication; history of neoadjuvant chemotherapy or radiation therapy; use of Enhanced Recovery after Surgery (ERAS) protocol | Randomized control trial (RCT) | - Postoperative exercise program - Twice-daily, 15-min/session - Supervised exercise, stretching, core exercise, resistance exercise, balance exercise and unsupervised walking - Low-to-moderate-intensity - Intervention duration depends on the hospital stay | Exercise group vs Usual care group | - Length of hospital stay - Time of flatus Anthropometrics measures included height, weight, and body composition - Functional outcome measures included function of lower extremities, balance ability, and functional capacity | - Low-to-moderate-intensity postsurgical exercise reduced length of hospital stay and the time to flatus - Improves bowel motility after colectomy procedure in patients with stages I-III colon cancer |
| 1. Arbane, G.   2014  London, UK(7) | - Sample size: 131 - Male:72, Female: 59 - Age: 67 ± 11 years old in intervention group, 68 ± 11 years old in control group - Adults with NSCLC referred for lung resection (lobectomy and/or pneumonectomy) via open thoracotomy or video-assisted thoracoscopic surgery (VATS) - Exclusion: (1) received exploratory surgery and were subsequently considered unsuitable for resection; (2) required admission to the intensive care unit >48 hours after surgery; or (3) received >72 hours of supplementary oxygen at rest to maintain oxy- gen saturation >90% | Multicenter Randomized control trial (RCT) | - Once-daily cycle (30-min/session), 4-week - Cycle component with the intensity a maximum of 60% to 90% of heart rate reserve - Strength training - Strength training based on the 10 maximum REP principle using appropriate - A home walking program after discharge | Exercise program group vs Usual care group | - Physical activity - Exercise tolerance-Incremental Shuttle Walk Distance (ISWT); 10-m walking test - Quadriceps muscle strength - Health-related quality of life - Length of stay and postoperative complications | - Regardless of group allocation, the patients had recovered their pre-operative exercise tolerance levels by 4-week after surgery - No significant differences in other measurements between the groups |
| 1. Kuehr, L.   2014  Heidelberg(8) | - Sample size: 40 - Male: 24, Female: 16 - Age: 60 ± 12 years old - Patients with histologically confirmed NSCLC undergoing radio-and/or chemotherapy - Inclusion: patients were approached before the initiation of chemotherapy (in metastatic disease not beyond third line) or radiotherapy; body mass index (BMI) > 18 kg.m^-2^; ability to follow the German study instructions and questionnaires - Exclusion: suffering from acute infectious diseases; inability to stand or walk; immobility lasting longer than two days; bone metastasis in the spine; sever neurologic disorders; serous cardiovascular diseases, grave pulmonary or renal insufficiency; addiction to alcohol and drugs or substance abuse in general | Prospective pilot study | - An 8-week consecutive hospital- and home -based combined endurance and resistance training program - Inpatient setting, 5-times per-week; outpatient setting, three times per-week - Brisk walks outside or inside the clinic with the possibility; different gymnastic exercise with or without dumbbells and a set of color-coded stretch bands - Borg scale target score: 12~14 for endurance and 14~16 for resistance exercises | None | - Feasibility (adequate adherence) - Functional capacity: Endurance-6-min walk test (6MWT); Strength-handheld dynamometry (HHDM) capacity - Patient-reported outcomes (questionnaire): QoL; Fatigue; Depression (Functional Assessment of Cancer Therapy-Lung (FACT-L), Multidimensional Fatigue Inventory (MFI), Patient Health Questionnaire (PHQ-9) | - Study showed significant improvements in both endurance and isometric muscle strength. - The participant (55%) were able to perform two or more training units per-week, and no adverse events related to the training were observed. - This study reveals that physical function remained at higher levels toward the follow-up; at the same time, quality of life declined nonsignificant. The increased physical function levels may suggest longer-term sustainability of the intervention |
| 1. Oechsle,K.   2014  Hamburg-Eppendorf, Germany(9) | - Sample size: 58 - Male: 34, Female: 14 (completed) - Age: 51.7 ± 13.3 years old in intervention group, 52.9 ± 15.4 years old in control group - Patients with acute myeloid leukemia undergoing myeloablative chemotherapy and high-dose chemotherapy with autologous peripheral blood stem cell transplantation for malignant hematologic diseases or solid tumors - Inclusion: stable clinical overall condition - Exclusion: symptomatic cardiovascular diseases; tumor infiltration of the skeletal system with risk of pathologic fractures or compression of the spinal cord; epilepsies; rheumatologic diseases; malnutrition (body mass index (BMI) < 18; obesity (BIM >30); insufficient cognitive function or inadequate knowledge of German language for questionnaire analysis - Suspend training when patients experienced fever >38.0 ^o^C, infection, platelet count < 20,000/μl, hemoglobin < 8 g/dl, higher grade cardiac arrhythmias, or life-threatening clinical complications (e.g., admission to the intensive care unit) | Randomized control trial (RCT) | - Supervised exercise program - Warm-up; bicycle ergometer endurance training for at least 10-min and up to 20-min; three resistance exercises using the main muscle groups for 20-min - Five-time per-week - Intensity of the ergometer training was individually adjusted based on the results of spiroergometry after randomization and prior to the first training - Strength training were performed for up to 20-min at 40~60% of their estimated one repetition maximum with two sets of 16-25 repetitions - Median duration was 21 days | Training group vs Control group | - Physical performance - Lung function, muscular strength, and subjectively perceived exertion - Quality of life and fatigue - Additional supplementary parameters included Karnofsky performance status, body weight, BMI, days of inpatient hospital stay, and the number of red blood cell or platelet transfusions - One year after completion of the study intervention, all patients were contacted by phone and interviewed on their overall condition | - Multimodal exercise including endurance and strength training has positive effects on physical performance, physical functioning, and treatment-related symptoms. - Especially for fatigue, nausea and emesis, in patients undergoing myeloablative chemotherapy with autologous stem cell transplantation |
| 1. Travier, N.   2015  Netherlands(10) | - Sample size: 204 - Sex: Mix - Age: 49.7 ± 8.2 years old in intervention group, 49.5 ± 7.9 years old in control group - The patients who full histological breast cancer diagnosis <6-week before recruitment - Inclusion: stage M0; no distant metastasis; scheduled for chemotherapy; not treated for any cancer in the preceding five years (except basal skin cancer); able to read and understand the Dutch language; Karnovsky Performance Status of ≥ 60; no contra-indications for physical activity - The 6-week period was extended to 10-week if patients had a mastectomy with immediate reconstruction involving the use of tissue expander - All patients participated in the 18-week exercise program during (part of their) chemotherapy | Two-arm Randomized control trial (RCT) | - Two aerobic and strength exercise sessions per-week - 60-min exercise classes, 18-week - The exercise program was individualized to the patients ‘preferences at the pretest - Intensity of the interval aerobic training was based on the heart rate at the ventilatory threshold as determined - Muscle strength training was performed for all major muscle groups; intensity was re-evaluated weeks every 4-week | Exercise intervention group vs Control group | - Fatigue - Quality of life - Aerobic capacity - Thigh muscle strength - Handgrip strength - Body weight and height - Physical activity level | - The 18-week supervised exercise intervention reduces short-term physical fatigue and diminishment of cardiorespiratory fitness and improved muscle strength. - At 36-week later, effects were no longer statically significant, probably due to participants’ high activity levels during follow-up |
| 1. Fiuza-Luces, C.   2017  Madrid, Spain(11) | - Sample size: 49 - Male: 35, Female: 14 - Age: 10 ± 1 years old in intervention group, 11 ± 1 years old in control group - Pediatric cancer patients with solid tumors, not having received previous therapy other than surgery - Good performance status, for patients age ≥ 12 years old, score ≤ 2 on the Eastern Cooperative Oncology Group scale, and for patients age ≤ 12 years old, score ≥ 50% on the Lansky scale | Randomized control trial (RCT) | - Three sessions per-week (Monday-Wednesday-Friday), each session lasting 60~70-min - 19 ± 2 weeks - Aerobic exercise - Strength training | Intervention group vs Control group | - Muscle strength - Cardiorespiratory fitness - Body mass and BMI - Functional capacity in ADL - PA levels-accelerometry - Quality of life | - An in-hospital exercise program for pediatric cancer patients with solid tumors undergoing neoadjuvant treatment can be safely applied to increases upper and lower-body muscle strength despite the aggressiveness of such therapy |
| 1. Fox, L.   2017  London, UK(12) | - Sample size: 76 (not clear for the sample size information) - Sex: Male - Age: 63.63 ± 9.24 years old - The man with either localized or advanced prostate cancer (PCa) | Free-response telephone survey | - Once a week for eight sessions over a 10-week period (allowing for the patient to miss up to sessions) - 60-mins session, combined circuit, of aerobic and progressive resistive exercise; additional tailored exercise such as pelvic floor muscle exercise was prescribed as per individual need - The workload was defined by the heart rate reserve (HRR) (aerobic exercise) and one-repetition maximum (1-RM) measurement for resistance exercise - Combined a home walking exercise program | None | - Physiotherapy guidance - Structured classes as a motivator - Behavior changes - Social support - Individual differences | - Design of a structured exercise intervention for patients with prostate cancer (PCa) should embrace the positive aspects outlined here but consider patients individual differences - Ongoing feedback from patients should be utilized alongside traditional study designs to inform intervention designs in this area |
| 1. Leak Bryant, A   2017  Carolina(13) | - Sample size: 9 - Male: 5, Female: 1 (completed) - Age: (Mean) 55 years old - Inclusion: aged 21 years or older; being newly diagnosed with Acute myelogenous leukemia (AML) or acute lymphocytic leukemia (ALL); receiving induction therapy to begin chemotherapy with an expected hospital stay of 3~4 weeks; being able to speak and understand English - Exclusion: cardiovascular disease; acute or chronic respiratory disease; acute or chronic bone, muscle, or joint abnormalities; altered mental state, dementia, or any other psychological condition that would prevent understanding of informed consent; another active malignancy; active bleeding, acute thrombosis, ischemia, hemodynamic instability, or uncontrolled pain | Semi-structured interview | - Aerobic (walking or stationary bike) and resistance training (use of different strengths of resistance bands) - Progressive exercise model consisted of aerobic training for 5~15 min and resistance training for 10~30-min - Twice per-day, four times a week - The average length of stay in-hospital was 31 days | Exercise intervention group vs Control group | - Pre-diagnosis exercise activity - Perceived benefits of exercise - Perceived barriers of exercise - Post-hospital plan and resources - Praise for the overall program - Suggestions for program | - Patients were highly pleased with the exercise intervention and overall program - Common barriers to exercise were anxiety and aches and pains - Overall, participants experience physical and psychological benefits with the exercise intervention with no adverse events from exercising regularly during induction chemotherapy |
| 1. Platschek, A. M.   2017  German(14) | - Sample size: 9 - Male: 3, Female: 6 - Age: 11.33 ± 2.24 years old - Cancer patients with cancer; leukemia n=3, lymphoma n=3, sarcoma n=2, and neuroblastoma n=1 - Exclusion: age younger than 6 years or older than 18 years old; difficulty performing exercise sessions because of general physical or cognitive impairments; lack of language | Pilot study | - Computer-Based exercise intervention - 12-week; each session about 45-min - Participants could choose between a fitness and gymnastics game, a sports game, a dance game, and three other adventure games - The game offered different exercise categories and difficulty levels and included aspects of endurance, strength, coordination, and dexterity - The intensity and duration of each intervention session were self-selected and chosen depending on the patient’s age, individual aerobic capacity, and daily condition, as well as whether the patient was inpatient or outpatient | None | - Mood-modified paper-and-pencil MoodMeter ® questionnaire had two scales that assessed physical well-being and perceived psychological strain before and after the exercise intervention at weeks four, eight, and 12; - Fatigue-German version of PedsQLTM Multidimensional Fatigue Scale at baseline and at weeks six and 12 | - The intervention was feasible and provide preliminary evidence for the benefits on mood and fatigue in pediatric with cancer |
| 1. Morales, J. S.   2018  Madrid(15) | - Sample size: 49 - Male: 17, Female: 7 (completed) - Age: 10 ± 4 years old - Children with solid tumors | Randomized control trial (RCT) | - Training program which took place during the entire neoadjuvant chemotherapy treatment period, 19 ± 8-week - Three sessions per-week, each lasting ~60~70-min - Aerobic exercise; training load was gradually increased depending on the age, physical capacity and health status of child; exercise intensity was 60~70% of the maximum HR value - Strength training; the load was gradually increased as the strength of each child improved | Exercise intervention group vs Control group | - Muscle strength - Functional mobility - Cardiorespiratory fitness (CRF): peak oxygen uptake (VO_2peak_) | - In-hospital exercise interventions in children with solid tumors undergoing neoadjuvant treatment improved muscle strength safety - A considerable individual variability was observed for the improvements in functional mobility and cardiorespiratory fitness (CRF) |
| 1. Schram, A.   2019  Canada(16) | - Sample size: 30 - Male: 20, Female: 10 - Age: 63.4 ± 12.7 years old - Patients scheduled for curative colorectal resection - Exclusion: known metastases; contraindications to exercise and the inability to communicate in either English or French | A feasibility study | - Immediate post-operative period-the resistance exercise program consisted of a who body workout targeting all major muscle groups; the team kinesiologist would adjust the training load and intensity according to the patient’s situation; standard enhanced recovery after surgery protocols (ERAS) care - Post-operative period, 4-week intervention - Upon discharge, patients were instructed to continue their in-hospital resistance training program, as well as the instruction to accumulate a minimum of 30-min of activity per-day | None | - Primary outcomes: compliance to in-hospital resistance training - Secondary outcomes: enhanced recovery after surgery protocols (ERAS) guidelines for early mobilization and quality of recovery | - The implementation of an individualized and stratified resistance training program is feasible, safe and provides clinicians with an alternate means of encouraging in-hospital resistance training - Mitigated post-surgical functional decline associated with prolonged bed rest |
| 1. Rutkowska, A.   2019  Katowice(17) | - Sample size: 40 - Male: 27, Female: 3 (completed) - Age: 59.1 ± 6.8 years old in intervention group, 61.3 ± 8.8 years old in control group - Patients diagnosed with non-small cell lung cancer (NSCLC) at stage IIIB or IV, who were disqualified from surgery - Inclusion: the diagnosis was established with 6-week prior to enrollment and was confirmed by histology; had the ability to perform the 6-min walk test (6MWT); were World Health Organization performance status 0~1; were able to complete questionnaires; had the willingness to participate in an exercise training program - Exclusion: had uncontrolled hypertension or unstable coronary artery disease; anemia (hemoglobin <10 g/dL); sever osteoarthritis; bone or central nervous system metastases | Randomized control trial (RCT) | - Rehabilitation program in chronic obstructive pulmonary disease (COPD) - 4-week exercise intervention performed in 2-week cycles interspersed with consecutive rounds of chemotherapy - Intensities based on initial 6-min walk test (6MWT) and spirometry assessments - 5-time/week exercise sessions; consisted of fitness and respiratory exercise, cycle ergometer or treadmill, resistance exercise, Nordic walking (totally more than two hours.) - Heart rate, oxygen saturation, and blood pressure was monitored | Exercise training group vs Control group | - Exercise performance by the Endurance-6-min walk test (6MWT) distance - Forced expiratory volume in 1 sec (FEV_1_) - Forced vital capacity (FVC) - The ratio of the FEV_1_/ FVC - Dyspnea - Functional fitness | - Planned, individualized and supervised exercise programs in patients with advanced lung cancer during chemotherapy are a practical and beneficial intervention for enhancing mobility and physical fitness |
| 1. Morales, J. S.   2020  Madrid, Spain(18) | - Sample size: 169 - Male: 109, Female: 60 - Age: 11 ± 3 years old in intervention group, 11 ± 4 years old in control group - Inclusion: aged 4~18 years old; new diagnosis of childhood cancer; diagnosed, treated, and followed at the Hospital Infantil Universitario Niño Jesús (HIUNJ); not having participated in intervention trials outside standard care | Prospective cohort study | - Aerobic and resistance exercise was performed during the entire neoadjuvant or intense chemotherapy treatment periods - 2~3 sessions/week on non-consecutive week-days, of ~60~70-min, median duration was 22-week - Aerobic part lasted 30~40-min, the training load was gradually increased depending on the age, physical capacity, and health status of each child; exercise intensity was monitored by 65%~80% of HR reserve - Resistance training part lasted ~30-min, the load was gradually increased (5%~10%) as the strength of each child improved and independently for each exercise | None | - Primary outcomes: data on mortality, relapse, metastasis, and length of hospitalization; the economic cost of hospitalization - Secondary outcomes: laboratory variables and glucose concentration, body mass index; left ventricular systolic function and fractional shortening | - Supervised in-hospital exercise is safe and plays a cardioprotective role during the neoadjuvant chemotherapy period for solid tumors or intensive chemotherapy for children with leukemias - Reduced hospitalization time, and therefore alleviating the economic burden |
| 1. Dennett, A. M.   2021  Melbourne, Australia(19) | - Sample size: 73 - Male: 41, Female: 32 - Age: 63 ± 11 years old - Patients were to be adult cancer survivors currently receiving or preparing for cancer treatment (curative or palliative intent) admitted as an inpatient or outpatient; had medical approval to exercise from their specialist or general practitioner - Exclusion: with a cognitive impairment or receiving end of life care | A prospective, pre-post study | - An exercise-based cancer rehabilitation program, individually tailored, supervised group-based circuit exercise class (60-min) once or twice weekly - Or a tailored home exercise program (30-min) for 8-week; participants opting received an initial face-to-face assessment only and an 8-week follow-up in-person or via telephone - Aerobic exercise combines with resistance exercise for two group | Hospital based program vs Home exercise program | - Demand - Acceptability - Implementation - Practicality - Limited efficacy | - This study demonstrated implementation a co-located, exercise-based rehabilitation program in a cancer unit is safe and feasible. Clinical gains in the study reinforce exercise interventions can be readily translated pragmatically into hospital settings - Co-location may improve access to exercise for cancer survivors - However, readiness to participate in rehabilitation during treatment needs to be considered by using flexible rehabilitation models to overcome issues with uptake and adherence |
| 1. Park, J. H.   2021  South Korea(20) | - Sample size: 19 - Male: 10, Female: 9 - Age: (Mean) 60 years old - Participants had a plan to receive first-line chemotherapy for metastatic solid cancer - Inclusion: age ≥ 20 years old; Eastern Cooperative Oncology Group (ECOG) performance status ≤ 2; life expectancy ≥ 4 months - Exclusion: brain metastases; bone metastases with a high risk of fracture; musculoskeletal disorders that inhibit participants from exercise; symptomatic heart disease including congestive heart failure, arrhythmia, or myocardial infraction diagnosed within the last six months; uncontrolled hypertension | A pilot study | - Exercise intervention involved hospital-based and home-based exercise over 12-week - Twice a week, aerobic exercise (cycle ergometry, 60~85% of maximum heart rate and lasted 30-min) and resistance exercise (nine types of bodyweight exercise, three sets of ten repetitions) in the hospital setting - The home-based exercise consisted of walking and nine resistance exercise three time a week | None | - Primary outcome were safety and feasibility of the exercise intervention - Secondary outcome measures were skeletal muscle mass and strength, functional capacity, Quality of life, and fatigue | - Exercise intervention are feasible and safe in patients with metastatic cancer - Exercise intervention can improve quality of life and prevent skeletal muscle loss during palliative chemotherapy |
| 1. Spreafico, F.   2021  Italy(21) | - Sample size: 44 - Male: 24, Female: 20 - Age: (Mean) 15.5 years old - Patients with solid tumors or lymphomas who were receiving or had completed their treatment - Inclusion: aged 5~21 years; no disabilities and/or morbidities sever enough to prevent any form physical activity | Experiment design | - Combinations of cardiovascular training, strength and endurance exercise, relaxation, and muscle stretching - Individual and lasted one hour - Three time a week for a total of 6-week - The exercises were prescribed by sports professional to suit patients’ capabilities, limitations and preferences | Engaged in the exercise program (GYM) vs Non-GYM | - Certified questionnaires on Quality of life and fatigue | - Exercise improves the satisfaction of children and adolescents with cancer with their physical, mental and social functioning |
| 1. Kirca, K.   2021  Turkey(22) | - Sample size: 49 - Male: 35, Female: 14 - Age: 10 ± 1 years old in intervention group, 11 ± 1 years old in control group - Patient with lung cancer - Inclusion: received at least one course of chemotherapy; scheduled to undergo at least three cycle of chemotherapy in the same center; enrolled in a treatment protocol in chemotherapy every 21 days; aged older than 18 years; absence of any psychiatric, neurological or muscular disease impairing cognitive function or altering the perception of reality; status of being literate; possessed functional performance; exhibited vital signs within normal ranges; possessed a phone; and willingness to participate in the study - Exclusion: receiving simultaneous chemoradiotherapy; having hearing and speaking problems while talking on the phone | Sigle-center Randomized control trial (RCT) | - Progressive relaxation exercises (PREs) - The relaxation exercises targeted the hands, upper arms, shoulder, face, chest, abdomen, hip, and upper and lower legs with MP3 recording - 10-min description of relaxation and followed by 30-min of relaxation instruction and 30-min of music - Patients informed of the need to continue the relaxation exercises when at home - 30-min at least once a day, every day | Experimental group vs Control group | - Memorial Symptom Assessment Scale (MSAS) - Strategies Used by people to Promote Health (SUPPH) scale | - Progressive relaxation exercises were potentially effective in promoting symptom management and improving the level of self-efficacy |
| 1. Mikkelsen, M. K.   2022  Denmark(23) | - Sample size: 20 - Male: 9, Female: 9 (completed) - Age: (Mean) 71 years old - Patients with diagnosis of advanced pancreatic cancer (PC), biliary tract cancer (BTC), non-small cell lung cancer (NSCLC) within three months - Inclusion: treatment with palliative systemic therapy; age 65 years or older - Exclusion: physical or mental conditions that prevented participation based on safety concerns; inability to speak Danish | A qualitative explorative study | - Team-based exercise twice weekly, 12-week - A protein drink after each exercise session - A home-based walking program using pedometers and individualized step-count goals - Supportive and individualized nurse-led counseling based on identified needs | Intervention standard treatment +multimodal intervention group vs Control standard treatment group | - Motivated to strengthen body and mind   Doing what only I can do   - Reaching goals with support from healthcare professionals and peers - Exercise as an integrated part of the treatment course - Overcoming undeniable physical limitations | - The participants experienced several benefits from participation, including physical improvements, increased energy, reduction of symptoms, and improved social engagement - Goal setting, being positively pushed and cheered on, and integration of fun games increased motivation - In contrast, being pushed beyond physical limitations and experiencing severe symptoms were experienced as barriers toward exercising |
| 1. Borges, R. C.   2014  Brazil(24) | - Sample size: 46 - Male: 18, Female: 11 (completed) - Age: 64.1 ± 12.5 years old in intervention group, 67.8 ± 9 years old in control group - chronic obstructive pulmonary disease (COPD) patients who had been hospitalized due to disease exacerbation - Inclusion: COPD (FEV1/FVC<70%) exacerbation characterized by an increase in sputum or cough or worsening of dyspnea; no hospitalization in the last 30 days; absence of musculoskeletal or neurological conditions; no participation in a program in the last 6 months; absence of any pulmonary diseases - Exclusion: patients transferred to the ICU before the 2^nd^ day of hospitalization; patients exhibiting changes in mental status; worsening of hypoxemia (PaO_2_<40 mmHg at room air) and/or respiratory acidosis (pH<7.25); hospitalization time less than 5 days; inability to complete any of the evaluations | Randomized control trial (RCT) | - Weightlifting exercises for six muscle groups in the upper and lower limbs (two sets of eight repetitions each) - Initial load set at 80% of the one-repetition maximum load - Intervention duration depends on the hospital stay | Training group vs Control group | - Endurance-6-min walk test (6MWT) - Health-related quality of life (HRQL) - Upper and lower limb muscle strength - Systemic inflammatory levels and blood gas analysis - Physical activity in daily life (PADL) - Lung function | - The resistance training during hospitalization improves Endurance-6-min walk test, health-related quality of life (HRQL) and lower limb muscle strength and without altering the levels of systemic inflammation - No change the level of physical activity in daily life (PADL) in the hospital or at home |
| 1. Torres-Sanchez, l.   2017  Granada, Spain(25) | - Sample size: 58 - Male: 44; Female:16 (the author had an error in reporting the data) - Age: 75.65 ± 6.25 years old in intervention group, 72.12 ± 8.19 years old in control group - Older patients due to an exacerbation of chronic obstructive pulmonary disease (COPD) were recruited - Inclusion: being clinically diagnosed with an acute exacerbation of COPD according to the criteria of the American Thoracic Society (ATS); being aged 65 years or older; patients who scored over three in the Brief Frailty Index - Exclusion: inability to provide informed consent; presence of psychiatric or cognitive disorders, progressive neurological or musculoskeletal disorders, severe orthopedic problems, organ failure, cancer, or inability to cooperate; patients who had experienced another exacerbation of COPD in the previous month; did not complete at least four days of intervention | Randomized, single-blind clinical trial | - Cycling exercise intervention using a pedal exerciser in addition to standard care (patients were sitting on a chair in a comfortable position during the biking) - The intervention was performed from the second day of admission to discharge - The duration depended on the length of hospital stay of each patient - Cycling time, velocity, and intensity were adapted to the patients’ levels of dyspnea and fatigue - The activity was stopped if the patient reached level six of dyspnea or fatigue in the Borg scale | Exercise intervention group vs Control group | - Lower-limb strength - Balance - Exercise capacity - For descriptive purpose, anthropometric measures, functionality, quality of life, physical activity levels, respiratory function, rest oxygen saturation (SpO_2_), percentages of forced vital capacity (%FEV), forced expiratory volume in the first second (%FEV^1^), dyspnea perception at rest was recorded | - An exercise intervention using a pedal exercise during the hospital stay of frail elderly patients with an acute exacerbation of chronic obstructive pulmonary disease (AECOPD) improved muscle strength, balance, and exercise capacity |
| 1. Yilmaz, F. T.   2018  Sivas, Turkey(26) | - Sample size: 64 - Male: 45, Female: 5 (completed) - Age (average): 61 years old in normal weight group, 65 years old in overweight group, 66 years old in obese group - Patients who had at least 1 year of diagnosis of stage I or II chronic obstructive pulmonary disease (COPD) according to the Global Initiative for Chronic Obstructive Lung Disease (GOLD) classification system; - Inclusion: clinically stable; body mass index (BMI) of ≥ 18·5 kg/m2; were literate; had not participated in a regular exercise program and or pulmonary rehabilitation program within the previous six months; did not have an orthopedic disorder or lower limb amputee hat could prevent participation in an exercise program; volunteered to participate - Exclusion: patients who had respiratory tract infection or acute exacerbation within the last three months; received treatment with oral corticosteroids within the last 6 months; had a pulmonary disorder other than COPD; had a history of myocardial infraction, unstable angina, or stroke within the last four months; had cancer, uncontrolled hypertension, uncontrolled diabetes, an orthopedic disorder; had a history of hospitalization for long-term treatment in the past year; had seeing, hearing, and/or sensing problems; refused to participated | Experimental design | - Walking program - Walk at least 30-min every day - 16-week | Normal eight group vs Overweight group vs Obese group | - Patient assessment form - Patient monitoring and assessment form - Modified Medical Research Council (MMRC) Dyspnea Scale - St. George’s Respiratory Questionnaire (SGRQ) - Walking program implementation form - Pedometers | - In this study, all participants benefitted by reducing their dyspnea symptoms, increasing their walking duration, steps, and improving measures of quality of life - Obese participants in particular demonstrated improvements in pulmonary function |
| 1. Karstoft, K.   2014  Denmark(27) | - Sample size: 10 - Male 7; Female 3 - Age: 60.3 ± 2.3 years old - Diagnosed with type 2 diabetes mellitus - Exclusion: use of exogenous insulin; use of β-blocking agents; smoking; pregnancy; evidence of liver, renal, or cardiopulmonary disease; diseases contra-indicating physical activity | A crossover, control study (randomized order) | - Interval walking (IW, repeated cycles of 3-min of slow and fast walking; continuous walking (CW) - IW aiming at 54% and 89% of VO_2_peak; CW aiming at 73% VO_2_peak (walking speed was adjusted to ensure correct intensities - One-hour exercise intervention | Interval walking (IW) group vs Continuous walking (CW)group vs Control (CON) group | - Mean Oxygen consumption (VO2) - Mean Heart Rate - Mean rate of perceived exertion (RPE) - Mean walking speed - Blood lactate - Mixed-meal tolerance test-blood glucose concentrations; Endogenous rates of glucose appearance (RaENDO); exogenous rates of glucose appearance from the mixed-meal tolerance test MMTT (RaMMTT); rates of glucose disappearance (Rd); glucose metabolic clearance rate (MCR); Fasting serum insulin levels; Fasting plasma glucagon levels; the insulin to glucagon ratio - Free-living glycemia | - This study shown that an aerobic interval type exercise session improves both postprandial and free-living glycemic control in type 2 diabetes mellitus patients compared with an oxygen consumption- and time duration- matched continuous exercise session |
| 1. Kataoka, H.   2017  Japan(28) | - Sample size: 12 - Male 8; Female 4 - Age: 59.0 ± 11.6 years old - Patients with type 2 diabetes mellitus who were treated for glycemic control - Exclusion: severe cardiac or lung disease; acute or chronic musculoskeletal disorders; acute metabolic dysregulation; other neurological or endocrine disorders; a history of stroke; implanted with metal such as bolts and metallic prosthetic joints, placement of a stent or pacemaker; previous or current asymmetric proximal lower leg weakness and toe deformity or atrophy of foot muscles | Pilot study | - Exercise program included conventional aerobic exercise (bicycle ergometer) and four different toe resistance training exercises - Exercise load was set at moderate level - Exercise was performed for 30-min two hour after lunch, 20 times in each side with three sets every day for 2-week | None | - Toe pinch force - Muscle mass - Clinical and laboratory measurements: collected date for age, height, Body weight, body mass index (BMI), duration of type 2 diabetes mellitus, systolic blood pressure, diastolic blood pressure, heart rate, ankle-brachial index, cardio-ankle vascular index, medication and laboratory test results; fasting plasma glucose, hemoglobin A1c (HbA1c), total cholesterol, low-density lipoprotein cholesterol, high-density lipoprotein cholesterol, triglycerides, serum creatinine | - Two weeks of toe resistance training significant increased toe pinch force and toe muscle quality in patients with type 2 diabetes mellitus |

**References**

1. Weinstein AA, Chin LMK, Keyser RE, Kennedy M, Nathan SD, Woolstenhulme JG, et al. Effect of aerobic exercise training on fatigue and physical activity in patients with pulmonary arterial hypertension. Respir Med [Internet]. 2013;107(5):778–84. Available from: http://dx.doi.org/10.1016/j.rmed.2013.02.006

2. Liu J, Feng W, Zhou J, Huang F, Long L, Wang Y, et al. Effects of sling exercise therapy on balance, mobility, activities of daily living, quality of life and shoulder pain in stroke patients: a randomized controlled trial. Eur J Integr Med [Internet]. 2020;35(February):101077. Available from: https://doi.org/10.1016/j.eujim.2020.101077

3. Kenny M, Gilmartin J, Thompson C. Video-guided exercise after stroke: a feasibility randomised controlled trial. Physiother Theory Pract. 2020;1–12.

4. Mahmood W, Ahmed Burq HSI, Ehsan S, Sagheer B, Mahmood T. Effect of core stabilization exercises in addition to conventional therapy in improving trunk mobility, function, ambulation and quality of life in stroke patients: a randomized controlled trial. BMC Sports Sci Med Rehabil. 2022;14(1):1–9.

5. Hwang CL, Yu CJ, Shih JY, Yang PC, Wu YT. Effects of exercise training on exercise capacity in patients with non-small cell lung cancer receiving targeted therapy. Supportive Care in Cancer. 2012;20(12):3169–77.

6. Ahn KY, Hur H, Kim DH, Min J, Jeong DH, Chu SH, et al. The effects of inpatient exercise therapy on the length of hospital stay in stages I-III colon cancer patients: Randomized controlled trial. Int J Colorectal Dis. 2013;28(5):643–51.

7. Arbane G, Douiri A, Hart N, Hopkinson NS, Singh S, Speed C, et al. Effect of postoperative physical training on activity after curative surgery for non-small cell lung cancer: A multicentre randomised controlled trial. Physiotherapy (United Kingdom) [Internet]. 2014;100(2):100–7. Available from: http://dx.doi.org/10.1016/j.physio.2013.12.002

8. Kuehr L, Wiskemann J, Abel U, Ulrich CM, Hummler S, Thomas M. Exercise in patients with non-small cell lung cancer. Med Sci Sports Exerc. 2014;46(4):656–63.

9. Oechsle K, Aslan Z, Suesse Y, Jensen W, Bokemeyer C, de Wit M. Multimodal exercise training during myeloablative chemotherapy: A prospective randomized pilot trial. Supportive Care in Cancer. 2014;22(1):63–9.

10. Travier N, Velthuis MJ, Steins Bisschop CN, van den Buijs B, Monninkhof EM, Backx F, et al. Effects of an 18-week exercise programme started early during breast cancer treatment: A randomised controlled trial. BMC Med [Internet]. 2015;13(1):1–11. Available from: http://dx.doi.org/10.1186/s12916-015-0362-z

11. Fiuza-Luces C, Padilla JR, Soares-Miranda L, Santana-Sosa E, Quiroga J v., Santos-Lozano A, et al. Exercise Intervention in Pediatric Patients with Solid Tumors: The Physical Activity in Pediatric Cancer Trial. Med Sci Sports Exerc. 2017;49(2):223–30.

12. Fox L, Cahill F, Burgess C, Peat N, … SRB research, 2017 undefined. Real world evidence: a quantitative and qualitative glance at participant feedback from a free-response survey investigating experiences of a structured exercise. HindawiCom [Internet]. 2017;2017. Available from: https://www.hindawi.com/journals/bmri/2017/3507124/abs/

13. Bryant AL, Walton AML, Pergolotti M, Phillips B, Bailey C, Mayer DK, et al. Perceived benefts and barriers to exercise for recently treated adults with acute leukemia. Oncol Nurs Forum. 2017;44(4):413–20.

14. Platschek A maria, Kehe L, Abeln V, Berthold F, Simon T, Str HK. Computer-Based Exercise Program: Effects of a 12-Week Intervention on Mood and Fatigue in Pediatric Patients With Cancer. Oncology Nursing society. 2015;21(6):280–6.

15. Morales JS, Padilla JR, Valenzuela PL, Santana-Sosa E, Rincón-Castanedo C, Santos-Lozano A, et al. Inhospital exercise training in children with cancer: Does it work for all? Front Pediatr. 2018;6(December):1–8.

16. Schram A, Ferreira V, Minnella EM, Awasthi R, Carli F, Scheede-Bergdahl C. In-hospital resistance training to encourage early mobilization for enhanced recovery programs after colorectal cancer surgery: A feasibility study. European Journal of Surgical Oncology [Internet]. 2019;45(9):1592–7. Available from: https://doi.org/10.1016/j.ejso.2019.04.015

17. Rutkowska A, Jastrzebski D, Rutkowski S, Zebrowska A, Stanula A, Szczegielniak J, et al. Exercise Training in Patients With Non-Small Cell Lung Cancer During In-Hospital Chemotherapy Treatment: A RANDOMIZED CONTROLLED TRIAL. J Cardiopulm Rehabil Prev. 2019;39(2):127–33.

18. Morales JS, Santana-Sosa E, Santos-Lozano A, Baño-Rodrigo A, Valenzuela PL, Rincón-Castanedo C, et al. Inhospital exercise benefits in childhood cancer: A prospective cohort study. Scand J Med Sci Sports. 2020;30(1):126–34.

19. Dennett AM, Zappa B, Wong R, Ting SB, Williams K, Peiris CL. Bridging the gap: a pre-post feasibility study of embedding exercise therapy into a co-located cancer unit. Supportive Care in Cancer [Internet]. 2021;29(11):6701–11. Available from: https://doi.org/10.1007/s00520-021-06261-2

20. Park JH, Park KD, Kim JH, Kim YS, Kim EY, Ahn HK, et al. Resistance and aerobic exercise intervention during chemotherapy in patients with metastatic cancer: a pilot study in South Korea. Ann Palliat Med. 2021;10(10):10236–43.

21. Spreafico F, Barretta F, Murelli M, Chisari M, Gattuso G, Terenziani M, et al. Positive Impact of Organized Physical Exercise on Quality of Life and Fatigue in Children and Adolescents With Cancer. Front Pediatr. 2021;9(June):1–10.

22. Kırca K, Kutlutürkan S. The effect of progressive relaxation exercises on treatment-related symptoms and self-efficacy in patients with lung cancer receiving chemotherapy. Complement Ther Clin Pract. 2021;45(September).

23. Mikkelsen MK, Michelsen H, Nielsen DL, Vinther A, Lund CM, Jarden M. ‘Doing What only I Can Do’: Experiences from Participating in a Multimodal Exercise-Based Intervention in Older Patients with Advanced Cancer - A Qualitative Explorative Study. Cancer Nurs. 2022;45(2):E514–23.

24. Borges RC, Carvalho CR. Impact of resistance training in chronic obstructive pulmonary disease patients during periods of acute exacerbation. Arch Phys Med Rehabil [Internet]. 2014;95(9):1638–45. Available from: http://dx.doi.org/10.1016/j.apmr.2014.05.007

25. Torres-Sánchez I, Valenza MC, Cabrera-Martos I, López-Torres I, Benítez-Feliponi Á, Conde-Valero A. Effects of an Exercise Intervention in Frail Older Patients with Chronic Obstructive Pulmonary Disease Hospitalized due to an Exacerbation: A Randomized Controlled Trial. COPD: Journal of Chronic Obstructive Pulmonary Disease. 2017;14(1):37–42.

26. Yilmaz FT, Aydin HT. The effect of a regular walking program on dyspnoea severity and quality of life in normal weight, overweight, and obese patients with chronic obstructive pulmonary disease. Int J Nurs Pract. 2018;24(3):1–11.

27. Karstoft K, Christensen CS, Pedersen BK, Solomon TPJ. The acute effects of interval-Vs continuous-walking exercise on glycemic control in subjects with type 2 diabetes: A crossover, controlled study. Journal of Clinical Endocrinology and Metabolism. 2014;99(9):3334–42.

28. Kataoka H, Miyatake N, Kitayama N, Murao S, Tanaka S. A pilot study of short-term toe resistance training in patients with type 2 diabetes mellitus. Diabetol Int. 2017;8(4):392–6.
