# Supplementary material for: Integration of exercise prescription into medical provision as a treatment for non-communicable diseases: A scoping review
Source: Front Public Health. 2023 Jul 12;11:1126244. doi: 10.3389/fpubh.2023.1126244 (PMC10369190; doi:10.3389/fpubh.2023.1126244)
Supplement: Supplementary file 3 [file Table_3.DOCX]

**Table 3. Quantitative Summary of the Studies Characteristics**

|  | **Details** | **N** | **~%** |
| --- | --- | --- | --- |
| **Publication Year** | 2012-2016(1–9)  2016-2022(10–28) | 9  19 | 32%  68% |
| **Gender Characteristics of Sample Size** | Male  Female  Mix group  Total | 759  405  204  1368 | 55%  30%  15% |
| **Research Design** | Randomized control trial (RCT): (1–5,8–10,15,16,19,24,25,27,28)  Crossover, control study(6)  Pilot study(7,12,14,22)  Interview(11,13,26)  Experimental design(17,21,23)  Cohort study(20)  Feasibility study(18) | 15  1  4  3  3  1  1 | 54%  4%  14%  10%  10%  4%  4% |
| **Exercise Interventions Type** | Aerobic exercise(1–4,6–11,13,15–17,20–23,26,27)  Resistance exercise(2,4,5,7–13,16,18,20–23,25–27)  Balance exercise(2,26)  Core exercise(2)  Computer-based exercise intervention(14)  Sling exercise therapy(19)  Video-guided exercise(24)  Flexibility exercise(26)  Respiratory exercise(27)  Progressive relaxation exercises(28) | 20  19  2  1  1  1  1  1  1  1 | 71%  68%  7%  4%  4%  4%  4%  4%  4%  4% |
| **Non-communicable Diseases Categories** | - Cardiovascular diseases: pulmonary arterial hypertension (PAH)(3), Stroke(19,24,25) - Cancers: Lung(1,4,7,21,26–28), Colon(2), Acute myeloid leukaemia and solid tumours(8), Breast(9,21), Pediatric cancer(10,14,16,20,23), prostate cancer (PCa)(11), Colorectal(18), Lower and upper gastrointestinal tract (GI)(21), Gynaecological(21), Multiple myeloma(21), Lymphoma(21), Leukaemia(13,21), Renal(21), Other(21), Metastatic cancer(22), Pancreatic(26), Biliary Tract(26) - Respiratory diseases: chronic obstructive pulmonary disease (COPD)(5,15,17) - Diabetes: Type 2 diabetes(6,12) | 4  19  3  2 | 14%  68%  11%  7% |
| **Implementation of outcomes** | Positive outcomes: (1–3,5–8,10–28)  Negative outcomes: (4) | 27  1 | 96%  4% |

**References**

1. Hwang CL, Yu CJ, Shih JY, Yang PC, Wu YT. Effects of exercise training on exercise capacity in patients with non-small cell lung cancer receiving targeted therapy. Supportive Care in Cancer. 2012;20(12):3169–77.

2. Ahn KY, Hur H, Kim DH, Min J, Jeong DH, Chu SH, et al. The effects of inpatient exercise therapy on the length of hospital stay in stages I-III colon cancer patients: Randomized controlled trial. Int J Colorectal Dis. 2013;28(5):643–51.

3. Weinstein AA, Chin LMK, Keyser RE, Kennedy M, Nathan SD, Woolstenhulme JG, et al. Effect of aerobic exercise training on fatigue and physical activity in patients with pulmonary arterial hypertension. Respir Med [Internet]. 2013;107(5):778–84. Available from: http://dx.doi.org/10.1016/j.rmed.2013.02.006

4. Arbane G, Douiri A, Hart N, Hopkinson NS, Singh S, Speed C, et al. Effect of postoperative physical training on activity after curative surgery for non-small cell lung cancer: A multicentre randomised controlled trial. Physiotherapy (United Kingdom) [Internet]. 2014;100(2):100–7. Available from: http://dx.doi.org/10.1016/j.physio.2013.12.002

5. Borges RC, Carvalho CR. Impact of resistance training in chronic obstructive pulmonary disease patients during periods of acute exacerbation. Arch Phys Med Rehabil [Internet]. 2014;95(9):1638–45. Available from: http://dx.doi.org/10.1016/j.apmr.2014.05.007

6. Karstoft K, Christensen CS, Pedersen BK, Solomon TPJ. The acute effects of interval-Vs continuous-walking exercise on glycemic control in subjects with type 2 diabetes: A crossover, controlled study. Journal of Clinical Endocrinology and Metabolism. 2014;99(9):3334–42.

7. Kuehr L, Wiskemann J, Abel U, Ulrich CM, Hummler S, Thomas M. Exercise in patients with non-small cell lung cancer. Med Sci Sports Exerc. 2014;46(4):656–63.

8. Oechsle K, Aslan Z, Suesse Y, Jensen W, Bokemeyer C, de Wit M. Multimodal exercise training during myeloablative chemotherapy: A prospective randomized pilot trial. Supportive Care in Cancer. 2014;22(1):63–9.

9. Travier N, Velthuis MJ, Steins Bisschop CN, van den Buijs B, Monninkhof EM, Backx F, et al. Effects of an 18-week exercise programme started early during breast cancer treatment: A randomised controlled trial. BMC Med [Internet]. 2015;13(1):1–11. Available from: http://dx.doi.org/10.1186/s12916-015-0362-z

10. Fiuza-Luces C, Padilla JR, Soares-Miranda L, Santana-Sosa E, Quiroga J v., Santos-Lozano A, et al. Exercise Intervention in Pediatric Patients with Solid Tumors: The Physical Activity in Pediatric Cancer Trial. Med Sci Sports Exerc. 2017;49(2):223–30.

11. Fox L, Cahill F, Burgess C, Peat N, … SRB research, 2017 undefined. Real world evidence: a quantitative and qualitative glance at participant feedback from a free-response survey investigating experiences of a structured exercise. HindawiCom [Internet]. 2017;2017. Available from: https://www.hindawi.com/journals/bmri/2017/3507124/abs/

12. Kataoka H, Miyatake N, Kitayama N, Murao S, Tanaka S. A pilot study of short-term toe resistance training in patients with type 2 diabetes mellitus. Diabetol Int. 2017;8(4):392–6.

13. Bryant AL, Walton AML, Pergolotti M, Phillips B, Bailey C, Mayer DK, et al. Perceived benefts and barriers to exercise for recently treated adults with acute leukemia. Oncol Nurs Forum. 2017;44(4):413–20.

14. Platschek A maria, Kehe L, Abeln V, Berthold F, Simon T, Str HK. Computer-Based Exercise Program: Effects of a 12-Week Intervention on Mood and Fatigue in Pediatric Patients With Cancer. Oncology Nursing society. 2015;21(6):280–6.

15. Torres-Sánchez I, Valenza MC, Cabrera-Martos I, López-Torres I, Benítez-Feliponi Á, Conde-Valero A. Effects of an Exercise Intervention in Frail Older Patients with Chronic Obstructive Pulmonary Disease Hospitalized due to an Exacerbation: A Randomized Controlled Trial. COPD: Journal of Chronic Obstructive Pulmonary Disease. 2017;14(1):37–42.

16. Morales JS, Padilla JR, Valenzuela PL, Santana-Sosa E, Rincón-Castanedo C, Santos-Lozano A, et al. Inhospital exercise training in children with cancer: Does it work for all? Front Pediatr. 2018;6(December):1–8.

17. Yilmaz FT, Aydin HT. The effect of a regular walking program on dyspnoea severity and quality of life in normal weight, overweight, and obese patients with chronic obstructive pulmonary disease. Int J Nurs Pract. 2018;24(3):1–11.

18. Schram A, Ferreira V, Minnella EM, Awasthi R, Carli F, Scheede-Bergdahl C. In-hospital resistance training to encourage early mobilization for enhanced recovery programs after colorectal cancer surgery: A feasibility study. European Journal of Surgical Oncology [Internet]. 2019;45(9):1592–7. Available from: https://doi.org/10.1016/j.ejso.2019.04.015

19. Liu J, Feng W, Zhou J, Huang F, Long L, Wang Y, et al. Effects of sling exercise therapy on balance, mobility, activities of daily living, quality of life and shoulder pain in stroke patients: a randomized controlled trial. Eur J Integr Med [Internet]. 2020;35(February):101077. Available from: https://doi.org/10.1016/j.eujim.2020.101077

20. Morales JS, Santana-Sosa E, Santos-Lozano A, Baño-Rodrigo A, Valenzuela PL, Rincón-Castanedo C, et al. Inhospital exercise benefits in childhood cancer: A prospective cohort study. Scand J Med Sci Sports. 2020;30(1):126–34.

21. Dennett AM, Zappa B, Wong R, Ting SB, Williams K, Peiris CL. Bridging the gap: a pre-post feasibility study of embedding exercise therapy into a co-located cancer unit. Supportive Care in Cancer [Internet]. 2021;29(11):6701–11. Available from: https://doi.org/10.1007/s00520-021-06261-2

22. Park JH, Park KD, Kim JH, Kim YS, Kim EY, Ahn HK, et al. Resistance and aerobic exercise intervention during chemotherapy in patients with metastatic cancer: a pilot study in South Korea. Ann Palliat Med. 2021;10(10):10236–43.

23. Spreafico F, Barretta F, Murelli M, Chisari M, Gattuso G, Terenziani M, et al. Positive Impact of Organized Physical Exercise on Quality of Life and Fatigue in Children and Adolescents With Cancer. Front Pediatr. 2021;9(June):1–10.

24. Kenny M, Gilmartin J, Thompson C. Video-guided exercise after stroke: a feasibility randomised controlled trial. Physiother Theory Pract. 2020;1–12.

25. Mahmood W, Ahmed Burq HSI, Ehsan S, Sagheer B, Mahmood T. Effect of core stabilization exercises in addition to conventional therapy in improving trunk mobility, function, ambulation and quality of life in stroke patients: a randomized controlled trial. BMC Sports Sci Med Rehabil. 2022;14(1):1–9.

26. Mikkelsen MK, Michelsen H, Nielsen DL, Vinther A, Lund CM, Jarden M. ‘Doing What only I Can Do’: Experiences from Participating in a Multimodal Exercise-Based Intervention in Older Patients with Advanced Cancer - A Qualitative Explorative Study. Cancer Nurs. 2022;45(2):E514–23.

27. Rutkowska A, Jastrzebski D, Rutkowski S, Zebrowska A, Stanula A, Szczegielniak J, et al. Exercise Training in Patients With Non-Small Cell Lung Cancer During In-Hospital Chemotherapy Treatment: A RANDOMIZED CONTROLLED TRIAL. J Cardiopulm Rehabil Prev. 2019;39(2):127–33.

28. Kırca K, Kutlutürkan S. The effect of progressive relaxation exercises on treatment-related symptoms and self-efficacy in patients with lung cancer receiving chemotherapy. Complement Ther Clin Pract. 2021;45(September).
