# Supplementary material for: Integration of exercise prescription into medical provision as a treatment for non-communicable diseases: A scoping review
Source: Front Public Health. 2023 Jul 12;11:1126244. doi: 10.3389/fpubh.2023.1126244 (PMC10369190; doi:10.3389/fpubh.2023.1126244)
Supplement: Supplementary file 4 [file Table_4.DOCX]

**Table 4. Synthesis for the Relevant Information of Exercise Intervention in Inpatient and Outpatient Setting**

| **Citations** | **Exercise Guidelines** | **Recruitment and Adherence** | **Experimental Context** | **Exercise Interventions**  **Providers** | **The Provider’s Role in the Programmes?** | **Exercise specialist Yes/No/No information** | **Acceptability** | **Resources** |
| --- | --- | --- | --- | --- | --- | --- | --- | --- |
| 1. Weinstein, A. A.   2013  USA(1) | - The protocol was approved by the respective Institutional Review Boards of the collaborating institutions | - Recruited from local outpatient pulmonary hypertension and advanced lung disease clinics - Adherence rate was 86% - The participants’ attendance ranging between 24 and 30 sessions | Clinical pulmonary rehabilitation clinic | --- | --- | No information | - The regimen that was used was easily incorporated into an existing clinical pulmonary rehabilitation program - The exercise regimen that was easily incorporated into an existing clinical pulmonary rehabilitation program | - Fatigue Severity Scale (FSS) - Human Activity Profile (HAP) |
| 1. Liu, J.   2020  Hengyang, Hunan, China(2) | - Sling exercise therapy（SET）program | - Recruited from First Affiliated Hospital - Adherence rate was 100% | The first affiliated hospital of the University of South China | - Therapist | - Therapist preformed the exercise intervention | No | - High adherence | - Sling suspension equipment |
| 1. Kenny, M.   2022  UK(3) | - Video-guided exercise | - Recruited form three acute or rehabilitation pathway wards, consisting of two stroke unit and a general neuro-rehabilitation ward - Adherence rate was around 79% | A large teaching hospital | - Language therapist - Exercise therapists - Physiotherapist - Assessors - Researcher and a co-moderating experienced researcher | - Language therapist provided support for the patient with mild/moderate aphasia - Exercise therapists provided verbal prompts helped correct movement; also provided control group instruction - Physiotherapist judged the number of different exercises - Pairs of assessors who were experienced therapists, did outcome assessments - Researchers did the interview for qualitative data | Yes | - Recruitment was feasible | - Mobile tablet - Motor Status Scale (MSS) - General self-efficacy scale (GSE) |
| 1. Mahmood, W.   2022  Pakistan(4) | - Setting initial exercise prescription | - Recruited from Ibrahim polyclinic-shadman, Ch Muhammad Akram teaching hospital-Raiwind, Rasheed hospital-Defence - Adherence rate was 100% | Ibrahim polyclinic-Shadman, Ch Muhammad Akram teaching hospital-Raiwind, Rasheed hospital-Defence | - Research assistant | - Research assistant did the allocation process | No information | --- | - Trunk impairment Scale (TIS) - Stroke-specific quality of life scale (SS-QOL) |
| 1. Hwang, C.L.   2012  Taiwan(5) | - Setting initial exercise prescription - The program was adjusted by the physical therapist | - Recruited from the outpatient department of the National Taiwan University hospital - The mean adherence rate of the exercise group was 71.2% | Outpatient department of the National Taiwan University Hospital | - Physical therapist - researcher - Assessor | - Physical therapist supervised the exercise session; adjusted exercise programme - Researcher did the allocation process - Blinded assessor performed all tests | No | - Through the interactions with the study personnel and patient education, patients in the exercise group may learn to better cope with their symptoms | - Borg Rating of Perceived Exertion (RPE) |
| 2. Ahn,K.Y.  2013  Seoul, Korea(6) | - Setting initial exercise prescription | - Recruited from the Colorectal Cancer Clinic at the Severance Hospital - Adherence rate was 86% | Colorectal Cancer Clinic at the Severance Hospital, inpatient exercise programme | - Therapist - Nurses - Investigator - Hospital staff - Surgeon | - Therapist instructed and supervised the exercise intervention - Nurses, investigator, and hospital staff recorded some endpoints - Surgeon blinded assessed the primary endpoint | No | - For 30-day after hospital discharge, only one case of wound infection, and none of patients had to undergo a second surgery and readmitted to the hospital during the follow-up period | --- |
| 1. Arbane, G.   2014  London, UK(7) | - Setting initial exercise prescription - All protocols were approved by the Southeast London NHS Ethical Review Board | - Adherence rate was 60% at 4-week after surgery | Teaching hospital (Hospital plus home exercise programme) | - Researcher | - Researcher enrolled and assessed the participants | No information | --- | - Borg Rating of Perceived Exertion (RPE) - Borg CR10 Breathlessness Scale (BBS) - Pedometer - Act iWatch |
| 1. Kuehr, L.   2014  Heidelberg(8) | - Setting initial exercise prescription | - Adherence rate was 78% after the exercise intervention; 55% finished 8-week follow-up tests (and perform two or more training unites per week) | Thoracic Oncology Department in the Clinic of Thoracic Diseases of the University Hospital Heidelberg  (Inpatient and outpatient care) | - Oncologists - Exercise specialist | - Oncologists enrolled the participants - Exercise specialist provided guidance | Yes | - The present study shows that a hospital-and home-based exercise program is feasible for patients with non-small cell lung cancer (NSCLC) while undergoing anticancer treatment - Did not observe any exercise-related adverse events even in the unsupervised home-based training situation - Successful link the in-hospital health care with a home-based training that adherence of 77% as for the regular phone calls and detailed information about how to overcome training barriers. The exercise intervention results in a continued, more active lifestyle | - Treadmill - Cycle ergometer - Borg scale |
| 1. Oechsle,K.   2014  Hamburg-Eppendorf, Germany(9) | - Setting initial exercise prescription | - Recruited from the University Medical Center of Hamburg-Eppendorf - Adherence rate was 83% | University Medical Centre of Hamburg | --- | --- | No information | - No relevant complications related to the training program were observed | - Bicycle ergometer - Borg scale - European Organization for Research and Treatment of Cancer Quality of Life Questionnaire Core 30 (EORTC-QLQ-C30) - Modified Fatigue Impact Scale (MFIS) |
| 1. Travier, N.   2015  Netherlands(10) | - Setting initial exercise prescription | - Participants were invited by their clinician or oncological nurse during a regular out-patient clinic visit - Adherence rate was 83% | Study was conducted in seven hospitals (one academic and six general hospitals) | - Researchers - Physiotherapist - Fitness instructors with previous experience in training | - Researchers blinded assessed outcome measurements - Physiotherapist supervised the exercise intervention - Fitness instructor supervised and individualized exercise for each patient | Yes | - The trial showed that an exercise intervention offered in the daily clinical practice and starting early during adjuvant treatment is feasible and safe | - Borg scale - Short Questionnaire to Assess Health enhancing physical activity (SQUASH) - Multidimensional Fatigue Inventory (MFI) - Fatigue Quality List (FQL) - 30-item European Organization for Research and Treatment of Cancer Quality of Life Questionnaire C30 - 36-item Short Form Health Survey (SF-36) - Hospital Anxiety and Depression Scale |
| 1. Fiuza-Luces, C.   2017  Madrid, Spain(11) | - Setting initial exercise prescription | - Recruited from Children’s Hospital - Adherence rate was 100% | Children’s Hospital Nin ̃ o Jesu ́ s | - Researcher - Physician | - Researcher blinded assessed eligibility, baseline measures, and outcome assessments - Physician provided the recommendation for exercise participation | No | - In-hospital exercise program can be safely applied - This is a particular need for early targeted exercise programs in pediatric cancer not only because long-term impairment of childhood cancer survivors but also because this may be especially problematic in children since muscle strength is essential for health and growth as well as for leisure and play time with peers, and such activities are important for individual a social development | - Hospital gymnasium and the equipment |
| 1. Fox, L.   2017  London, UK(12) | - Exercise program protocol was modelled from existing published cancer exercise and UK physical activity recommendations | - Recommended by physicians - Adherence rate was around 54% | Guy’s Hospital (A large cancer center hospital programme) | - Physiotherapist - Researcher | - Physiotherapist supervised the exercise programme - Researcher conducted the interview and produced data summary | No | - Patients’ descriptions of the motivational force of the group setting attributed this to both peer support and friendly intragroup competition - The exercise class enabled patients to talk with another patient who had been through the class already and had lost weight and got fit | - Cross trainer, treadmill, arm cycle, static cycling, multigym, dumbbells - Borg scale |
| 1. Leak Bryant, A   2017  Carolina(13) | - Setting initial exercise prescription | - Inpatient in the hospital - Adherence rate was around 67% | Inpatient hematology/oncology unit at North Carolina Cancer Hospital | - Exercise sport science specialists - Investigator | - Exercise sport science specialists supervised the exercise intervention - Investigator monitored the laboratory values of participants and did interviews | Yes | - Finding inform the nurses’ role in encouraging and supporting adults with acute leukemia to exercise and be physically active during their hospitalization | - Stationary bike - Resistance bands |
| 1. Platschek, A. M.   2017  German(14) | - Active video game (Microsoft® Xbox 360 Kinect) | - Department of pediatric oncology at the University Hospital of Cologne - Adherence rate was 100% | Inpatient and outpatient ward in a single hospital | - Sport scientist (an exercise professional) | - Sport scientist supervised each exercise intervention | Yes | - No dropouts or adverse events were observed in this pilot study - Can potentially maintain or increase interest for hospitalize patients | - Active video game (Microsoft® Xbox 360 Kinect) - Modified paper-and-pencil MoodMeter® questionnaire - PedsQL^TM^ Multidimensional Fatigue Scale |
| 1. Morales, J. S.   2018  Madrid(15) | - Setting initial exercise prescription | - Recruited from inpatients - Adherence rate was around 49% | In hospital | - Clinician - Investigator | - Clinician provided the recommendations for exercise participation - Investigator measured outcomes | No | - No major adverse events or health-related issues attributable to the testing or training sessions were noted | - Hospital gymnasium - Metabolic cart |
| 1. Schram, A.   2019  Canada(16) | - Setting initial exercise prescription - Enhanced recovery after surgery protocols (ERAS) | - Recruited from the pre-operative clinic at the Montreal General Hospital - Adherence rate was around 97% | Pre-operative clinic at the Montreal General Hospital | - Kinesiologist - Physician - Nurse | - Kinesiologist taught the programme, assessed patients’ capacity, adjusted training intensity and supervised and encouraged the patients - Physician confirmed patient’s medical situation - Nurse did a pre-mobilization and checked any adverse symptoms | Yes | - Based on the compliance to in-hospital resistance exercise and absence of adverse events, initiating supervised post-operative resistance exercise in the immediate 24-h following colorectal cancer surgery is feasible and safe - Resistance training does not require the big space as is required for performing endurance-based exercise | - Borg scale - Resistance bands - MILES questionnaire |
| 1. Rutkowska, A.   2019  Katowice(17) | - Rehabilitation program in chronic obstructive pulmonary disease (COPD) | - Recruited from an Independent Public Clinical Hospital No. 3 of the Medical University of Silesia - Adherence rate was 75% | Independent Public Clinical Hospital No. 3 of the Medical University | - Physiotherapist - Doctors - Study coordinator | - Physiotherapist supervised the exercise programme, scheduled study, did the allocation process - Doctors enrolled, assigned, and supervised patients - Study coordinator assessed eligibility | No | --- | - Rehabilitation program in chronic obstructive pulmonary disease (COPD) - American Thoracic Society 2002 guidelines - European Respiratory Society recommendations - Modified Medical Research Council (mMRC) questionnaire - Baseline Borg dyspnea scale - Dyspnea Index (BDI) |
| 1. Morales, J. S.   2020  Madrid, Spain(18) | - The aerobic and resistance exercise protocol was tailored according to the previous guidelines for aerobic and strength training in children/adolescents | - Recruited from the public Hospital in Madrid - Adherence rate was 100% at post-treatment; ~90% at one year; ~69% at two years; ~53% at three years; ~32% at four years; ~17% at five years | Hospital Infantil Universitario Niño Jesús (HIUNJ) -a public hospital | - Physiologist (university-trained with a strong background in pediatric exercise) - Medical staff - Researcher | - Physiologist supervised the exercise sessions - Medical staff provided consultation for physiologist - Researcher collected data | Yes | - No major adverse events or health-related issues attributable to the training sessions were noted - The benefits on left ventricular function were not significantly maintained at one-year follow-up and thereafter significant declines shown in both groups compared to baseline at two and three-year follow-up that suggests exercise interventions should be prolonged to maximize their benefits | - Hospital exercise facility - Weight training machines specifically designed for children - Laboratory test equipment |
| 1. Dennett, A. M.   2021  Melbourne, Australia(19) | - Intervention description using the template for description and replication checklist (TIDier) - Setting initial exercise prescription | - Recruited from medical referrals, nursing referrals, allied health referrals, and self-referrals - Adherence rate was around 81% | Hospital-based cancer treatment unit | - Specialist or general practitioner - Physiotherapist | - Specialist approved the patient to conduct an exercise programme - Physiotherapist did the assessment and supervised the exercise class | No | - All participants reported they were satisfied with the program and would recommend it to others - Participants described physical and psychosocial benefits and highly valued staff - Participants had high confidence to continue exercising after the program finished according to the self-efficacy item on the survey - No major adverse event was recorded | - Hospital gymnasium: - Free weights - Resistance exercise bands - Pin-loaded machines (lateral pull down, leg press, chest press) - Aerobic exercise equipment (Included treadmill, walking, stationary cycle, arm ergometer, exercise pedals - Balance equipment - Borg Rating of Perceived Exertion (RPE) - Borg scale |
| 1. Park, J. H.   2021  South Korea(20) | - Setting initial exercise prescription | - Recruited from hospital - Adherence rate was around 71% - Mean attendance rate of the supervised exercise sessions was 64.9% | In hospital | - Accredited physical therapist | - Therapist supervised the exercise sessions, recorded any adverse event | Yes | - No adverse events or skeletal complications occurred during the supervised exercise sessions | - Cycle ergometry - computerized tomography (CT) - In-house software - European Organization for Research and Treatment of Cancer Quality of Life Questionnaire Core 30 (EORTC QLQ-C30) - Functional Assessment Cancer Therapy- General (FACT-G) - Functional Assessment of Chronic Illness Therapy-Fatigue Questionnaire (FACIT- Fatigue) |
| 1. Spreafico, F.   2021  Italy(21) | - Setting initial exercise prescription | - Recruited from hospital - Adherence rate was 100% | In hospital | - Sports professionals | - Sports professionals proscribed exercise that was suitable for the patients | Yes | - Even 5-years-olds children patients could take an active part in the workout sessions implemented in the present study | - Hospital gym - PedsQL-4.0 Generic Core Scales (Italian edition) - PedsQL Multidimensional Fatigue Scale (Italian edition) |
| 1. Kirca, K.   2021  Turkey(22) | - Progressive relaxation exercises protocol | - Recruited from Outpatient Chemotherapy Unit of Gazi University Health Research and Application Center - Adherence rate was around 86% | Outpatient Chemotherapy Unit of Gazi University Health Research and Application Center | - Clinic nurse - Investigators - A medical oncology specialist, nursing faculty members, medical oncology nurses, a radiation oncology nurse, education faculty lecturers - Assessor | - Nurse did the allocation process - Investigators provided educational booklet, demonstrated exercise performance, and evaluated patients in interview - A medical oncology specialist, nursing faculty members, medical oncology nurses, a radiation oncology nurse, education faculty lecturers provided consultation for investigator - Assessor made measurements | No | --- | - MP3 and earphones - Short message service - Counseling was provided weekly by Phone |
| 1. Mikkelsen, M. K.   2022  Denmark(23) | - Setting initial exercise prescription | - Recruited from Department of Oncology, Herlev and Gentofte Hospital, Denmark - Adherence rate was 90% | Department of Oncology, Herlev and Gentofte Hospital | - Experienced oncology research nurse | - Nurse contacted and scheduled interview, and did the interviews | No | - Several motivational factors that encouraged exercising were identified | - Pedometers |
| 1. Borges, R. C.   2014  Brazil(24) | - Setting initial exercise prescription | - Recruited from the hospital - Adherence rate was 63% | Ward of a University Hospital | - Evaluator - Investigator - Physiotherapist | - Evaluator evaluated patients’ performance - Investigator did the allocation process - Physiotherapist provided exercise intervention | No | - Whole-body resistance training (WBRT) proved to be safe and feasible to implement in exacerbated chronic obstructive pulmonary disease (COPD) patients admitted to a medical ward | - Free weights - Dumbbells - Borg dyspnea scores - Modified Borg scale |
| 1. Torres-Sanchez, l.   2017  Granada, Spain(25) | - Setting initial exercise prescription | - Recruited from the respiratory ward of San Cecilio and Virgen de las Nieves hospitals - Adherence rate is 100 % | Hospital | - Research assistant - Nurse - Physiotherapist - Researcher | - Research assistant collected the data - Nurse did the allocation process - Physiotherapist instructed the exercise programme - Researcher collected the data | No | - Handheld dynamometry had shown to be a feasible, inexpensive, and portable test of quadriceps muscle strength - No adverse effects were noted during the intervention sessions | - Barthel Index - Portable handheld dynamometer - St. George’s Respiratory Questionnaire - Modified Baecke physical activity questionnaire - Spirometry - Pulse oximeter - Borg scale |
| 1. Yilmaz, F. T.   2018  Sivas, Turkey(26) | - Setting initial exercise prescription | - Recruited from a respiratory clinic of a public hospital - Adherence rate is around 78% | Respiratory clinic of a public hospital | - Researchers - Nurse | - Researchers provided assessment forms for patients - Nurse involved in every implementation stage | No | - Chronic obstructive pulmonary disease (COPD) patient education clinic supervised by nurses could be run in health care institutions, with the purpose of improving activities of daily life by short-term walking programs | - Modified Medical Research Council (MMRC) Dyspnea Scale - St. George’s Respiratory Questionnaire (SGRQ) - Pedometers |
| 1. Karstoft, K.   2014  Denmark(27) | - Setting initial exercise prescription | - Adherence rate is 100% | In hospitalized and ambulatory care | --- | --- | No information | - These findings highlight the importance of considering exercise mode and not just exercise volume and mean intensity when implementing physical activity in diabetes care | - Actiheart; CamNtech-a triaxial accelerometer - CGM; Guardian Real-Time with Enlite glucose sensor; Medtronic - Cosmed; wireless HR monitor |
| 1. Kataoka, H.   2017  Japan(28) | - Setting initial exercise prescription | - Adherence rate is 100% | KKR Takamatsu Hospital | - Well-trained medical staff | - Medical staff interviewed each patient to evaluate the International Physical Activity Questionnaire, and did measurements | No | --- | - HBR-2070 (Omron Colin Co. Ltd., Tokyo, Japan) for blood pressure and heart rate measurement - VS-1500 (Fukuda Denshi Co. Ltd., Tokyo, Japan) for ankle-brachial index and cardio-ankle vascular index - Guidelines of the Japan Diabetes Society |

**References**

1. Weinstein AA, Chin LMK, Keyser RE, Kennedy M, Nathan SD, Woolstenhulme JG, et al. Effect of aerobic exercise training on fatigue and physical activity in patients with pulmonary arterial hypertension. Respir Med [Internet]. 2013;107(5):778–84. Available from: http://dx.doi.org/10.1016/j.rmed.2013.02.006

2. Liu J, Feng W, Zhou J, Huang F, Long L, Wang Y, et al. Effects of sling exercise therapy on balance, mobility, activities of daily living, quality of life and shoulder pain in stroke patients: a randomized controlled trial. Eur J Integr Med [Internet]. 2020;35(February):101077. Available from: https://doi.org/10.1016/j.eujim.2020.101077

3. Kenny M, Gilmartin J, Thompson C. Video-guided exercise after stroke: a feasibility randomised controlled trial. Physiother Theory Pract. 2020;1–12.

4. Mahmood W, Ahmed Burq HSI, Ehsan S, Sagheer B, Mahmood T. Effect of core stabilization exercises in addition to conventional therapy in improving trunk mobility, function, ambulation and quality of life in stroke patients: a randomized controlled trial. BMC Sports Sci Med Rehabil. 2022;14(1):1–9.

5. Hwang CL, Yu CJ, Shih JY, Yang PC, Wu YT. Effects of exercise training on exercise capacity in patients with non-small cell lung cancer receiving targeted therapy. Supportive Care in Cancer. 2012;20(12):3169–77.

6. Ahn KY, Hur H, Kim DH, Min J, Jeong DH, Chu SH, et al. The effects of inpatient exercise therapy on the length of hospital stay in stages I-III colon cancer patients: Randomized controlled trial. Int J Colorectal Dis. 2013;28(5):643–51.

7. Arbane G, Douiri A, Hart N, Hopkinson NS, Singh S, Speed C, et al. Effect of postoperative physical training on activity after curative surgery for non-small cell lung cancer: A multicentre randomised controlled trial. Physiotherapy (United Kingdom) [Internet]. 2014;100(2):100–7. Available from: http://dx.doi.org/10.1016/j.physio.2013.12.002

8. Kuehr L, Wiskemann J, Abel U, Ulrich CM, Hummler S, Thomas M. Exercise in patients with non-small cell lung cancer. Med Sci Sports Exerc. 2014;46(4):656–63.

9. Oechsle K, Aslan Z, Suesse Y, Jensen W, Bokemeyer C, de Wit M. Multimodal exercise training during myeloablative chemotherapy: A prospective randomized pilot trial. Supportive Care in Cancer. 2014;22(1):63–9.

10. Travier N, Velthuis MJ, Steins Bisschop CN, van den Buijs B, Monninkhof EM, Backx F, et al. Effects of an 18-week exercise programme started early during breast cancer treatment: A randomised controlled trial. BMC Med [Internet]. 2015;13(1):1–11. Available from: http://dx.doi.org/10.1186/s12916-015-0362-z

11. Fiuza-Luces C, Padilla JR, Soares-Miranda L, Santana-Sosa E, Quiroga J v., Santos-Lozano A, et al. Exercise Intervention in Pediatric Patients with Solid Tumors: The Physical Activity in Pediatric Cancer Trial. Med Sci Sports Exerc. 2017;49(2):223–30.

12. Fox L, Cahill F, Burgess C, Peat N, … SRB research, 2017 undefined. Real world evidence: a quantitative and qualitative glance at participant feedback from a free-response survey investigating experiences of a structured exercise. HindawiCom [Internet]. 2017;2017. Available from: https://www.hindawi.com/journals/bmri/2017/3507124/abs/

13. Bryant AL, Walton AML, Pergolotti M, Phillips B, Bailey C, Mayer DK, et al. Perceived benefts and barriers to exercise for recently treated adults with acute leukemia. Oncol Nurs Forum. 2017;44(4):413–20.

14. Platschek A maria, Kehe L, Abeln V, Berthold F, Simon T, Str HK. Computer-Based Exercise Program: Effects of a 12-Week Intervention on Mood and Fatigue in Pediatric Patients With Cancer. Oncology Nursing society. 2015;21(6):280–6.

15. Morales JS, Padilla JR, Valenzuela PL, Santana-Sosa E, Rincón-Castanedo C, Santos-Lozano A, et al. Inhospital exercise training in children with cancer: Does it work for all? Front Pediatr. 2018;6(December):1–8.

16. Schram A, Ferreira V, Minnella EM, Awasthi R, Carli F, Scheede-Bergdahl C. In-hospital resistance training to encourage early mobilization for enhanced recovery programs after colorectal cancer surgery: A feasibility study. European Journal of Surgical Oncology [Internet]. 2019;45(9):1592–7. Available from: https://doi.org/10.1016/j.ejso.2019.04.015

17. Rutkowska A, Jastrzebski D, Rutkowski S, Zebrowska A, Stanula A, Szczegielniak J, et al. Exercise Training in Patients With Non-Small Cell Lung Cancer During In-Hospital Chemotherapy Treatment: A RANDOMIZED CONTROLLED TRIAL. J Cardiopulm Rehabil Prev. 2019;39(2):127–33.

18. Morales JS, Santana-Sosa E, Santos-Lozano A, Baño-Rodrigo A, Valenzuela PL, Rincón-Castanedo C, et al. Inhospital exercise benefits in childhood cancer: A prospective cohort study. Scand J Med Sci Sports. 2020;30(1):126–34.

19. Dennett AM, Zappa B, Wong R, Ting SB, Williams K, Peiris CL. Bridging the gap: a pre-post feasibility study of embedding exercise therapy into a co-located cancer unit. Supportive Care in Cancer [Internet]. 2021;29(11):6701–11. Available from: https://doi.org/10.1007/s00520-021-06261-2

20. Park JH, Park KD, Kim JH, Kim YS, Kim EY, Ahn HK, et al. Resistance and aerobic exercise intervention during chemotherapy in patients with metastatic cancer: a pilot study in South Korea. Ann Palliat Med. 2021;10(10):10236–43.

21. Spreafico F, Barretta F, Murelli M, Chisari M, Gattuso G, Terenziani M, et al. Positive Impact of Organized Physical Exercise on Quality of Life and Fatigue in Children and Adolescents With Cancer. Front Pediatr. 2021;9(June):1–10.

22. Kırca K, Kutlutürkan S. The effect of progressive relaxation exercises on treatment-related symptoms and self-efficacy in patients with lung cancer receiving chemotherapy. Complement Ther Clin Pract. 2021;45(September).

23. Mikkelsen MK, Michelsen H, Nielsen DL, Vinther A, Lund CM, Jarden M. ‘Doing What only I Can Do’: Experiences from Participating in a Multimodal Exercise-Based Intervention in Older Patients with Advanced Cancer - A Qualitative Explorative Study. Cancer Nurs. 2022;45(2):E514–23.

24. Borges RC, Carvalho CR. Impact of resistance training in chronic obstructive pulmonary disease patients during periods of acute exacerbation. Arch Phys Med Rehabil [Internet]. 2014;95(9):1638–45. Available from: http://dx.doi.org/10.1016/j.apmr.2014.05.007

25. Torres-Sánchez I, Valenza MC, Cabrera-Martos I, López-Torres I, Benítez-Feliponi Á, Conde-Valero A. Effects of an Exercise Intervention in Frail Older Patients with Chronic Obstructive Pulmonary Disease Hospitalized due to an Exacerbation: A Randomized Controlled Trial. COPD: Journal of Chronic Obstructive Pulmonary Disease. 2017;14(1):37–42.

26. Yilmaz FT, Aydin HT. The effect of a regular walking program on dyspnoea severity and quality of life in normal weight, overweight, and obese patients with chronic obstructive pulmonary disease. Int J Nurs Pract. 2018;24(3):1–11.

27. Karstoft K, Christensen CS, Pedersen BK, Solomon TPJ. The acute effects of interval-Vs continuous-walking exercise on glycemic control in subjects with type 2 diabetes: A crossover, controlled study. Journal of Clinical Endocrinology and Metabolism. 2014;99(9):3334–42.

28. Kataoka H, Miyatake N, Kitayama N, Murao S, Tanaka S. A pilot study of short-term toe resistance training in patients with type 2 diabetes mellitus. Diabetol Int. 2017;8(4):392–6.
