# Supplementary material for: Integration of exercise prescription into medical provision as a treatment for non-communicable diseases: A scoping review
Source: Front Public Health. 2023 Jul 12;11:1126244. doi: 10.3389/fpubh.2023.1126244 (PMC10369190; doi:10.3389/fpubh.2023.1126244)
Supplement: Supplementary file 5 [file Table_5.DOCX]

**Table 5. Suggestions for Future Research Directions Based on the Current Evidence**

|  | **Existing Research** | **Research Limitation** | **Future Research Direction** |
| --- | --- | --- | --- |
| **Cardiovascular Diseases** | - To investigate the effectiveness of an exercise intervention for decreasing fatigue severity and increasing physical activity in individuals with pulmonary arterial hypertension (PAH)(1) - To explore the effectiveness of sling exercise therapy on balance, mobility, activities of daily living, quality of life and shoulder pain in stroke patients compared with routine physiotherapy(2) - To investigate the feasibility and acceptability of video-guided exercise for facilitating upper-limb exercise after stroke(3) - To determine the effectiveness of core stabilization exercises versus conventional therapy on trunk mobility, function, ambulation, and quality of life of stroke patients(4) | - Small sample size(1–4) - The result was self-reported(1) - Single sex participants(1) - Withdrew due to changes in medication(1) - Low attendance at the exercise sessions(1) - Performance and social desirability biases(3) - No sample sizes calculation(3) | - The long-term effects, durability and the feasibility of aerobic exercise training on PAH survival - Male participants research - The optimal duration and frequency of training - Additional studies on the effects of SET on functional recovery in stroke patients are required - Measure base-line cognitive functioning to isolate its role as potential effect moderator in the qualitative studies - Longer time period and extending the intervention into community setting - The effect of self-directed therapy - Long-term, larger sample size, and generalizability of exercise intervention |
| **Cancer** | - To evaluate the effect of a combined hospital plus home exercise program following curative surgery for non-small cell lung cancer (NSCLC)(5) - To investigate whether exercise training improves exercise capacity and other related factors in patients with NSCLC receiving targeted therapy(6–8) - To examine the effects of postsurgical, inpatient exercise program on postoperative recovery in operable colon cancer patients(9) - To evaluated the effects of multimodal aerobic and strength exercise on physical performance in hospitalized cancer patients while receiving myeloablative chemotherapy(10) - To examine the effects of an 18-week exercise intervention, offered in the daily clinical practice setting and starting within 6-week after diagnosis, on preventing an increase in fatigue with breast cancer(11) - To determine the effects of an in-hospital exercise intervention on pediatric cancer patients (with solid tumors undergoing neoadjuvant chemotherapy)(12–15) - To explore patients experiences of a structure exercise intervention for men with prostate cancer (PCa)(16) - To explore perceived exercise benefits and barriers in adults with acute leukemia who recently completed an inpatient exercise intervention during induction therapy(17) - To determine to benefits of a computer-based exercise intervention on perceived physical, motivational, and fatigue syndrome and psychological state in pediatric cancer patients(18) - To investigate the feasibility of initiating resistance exercise to promote early mobilization in colorectal cancer(19) - To establish the feasibility of embedding a flexible, exercise-based rehabilitation program into a cancer treatment unit to allow cancer (and/or metastatic) survivors early exercise support(20,21) - To explore the experiences of older patients with advanced cancer who participated in a 12-week multimodal exercise program(22) - To determine the effect of progressive relaxation exercise on treatment-related symptoms and self-efficacy in patients with lung cancer receiving chemotherapy(23) | - Small sample size(6,7,9,10,13,15,17,18,20,21) - Difficulty in recruiting participants(6,13) - Adherence problem(5–10,13,14,16,21,23) - Patients’ motivation(6) - Exercise study as unblinded(5,9,16) - Due to the timing and nature of surgery, the authors were unable to collect preoperative activity data(5) - Less-attendance(6,21) - Hospital policy changed(9) - The authors were unable to supervise some process(5,20,21) - Lack of control group(7,16,18,19,21) - Lack of assessment of psychosocial parameters(10) - The high level of physical activity reported by 56% of the controls have led to an underestimation of the true effect(11) - Outcome was assessed more descriptive instead of objective measurement and analytical(11,15–18,22,23) - Change some testing items as for the patients’ medical reason(12) - Familiar to the testing equipment lead the neural adaptation that increase the performance(12) - Heterogeneity in participants’ characteristics(13–15) - Non-randomized(14,15,20) - Lack of biochemical marker measurements(8) | - The underlying mechanisms of benefits of exercise - Measure the long-term postoperative complications after the exercise intervention - Test and determine optimal intensity, frequency and duration of exercise intervention - Elucidate patients’ attitude, motivation, and barriers towards participation in exercise programs in order to specifically design exercise programs for the less active patients - The effect and optimal dosage of exercise programs in the pediatric cancer - Qualitative combine with quantitative study and different ethnic groups - Use of wearable devices for patients-generated health data for assist them after discharge - Long-term follow-up are needed to determine and lasting benefits - If applying a higher training stimulus (higher intensity and/or volume) might maximize responsiveness in pediatric cancer - Future work to conducted in order to understand the barriers to increasing patient mobility using a multidisciplinary perspective in order to translate evidence into practice and improve patient outcomes - Determine if logger intervention induce additional, long-lasting benefits, notably in cardiac function - More research is required to confirm benefits to health services to drive policy change and fund exercise-rehabilitation programs as part of standard care - Appropriate qualifications for rehabilitation, including a functional assessment of the patient, contributed to the proper selection of load and intensity of training, and thereby to the choice of the appropriate model of rehabilitation according to the individual abilities of the patient |
| **Respiratory Diseases** | - To elevate the effect of whole-body resistance training on exercise capacity, Health-related quality of life (HRQL) and muscle strength in patients hospitalized for exacerbation of chronic obstructive pulmonary disease (COPD)(24) - To determine whether an exercise intervention using a pedal exerciser is able to reduce disability in frail older patients with acute exacerbation of chronic obstructive pulmonary disease (AECOPD) during hospitalization(25) - To determine the effects of the regular walking program on the body mass index, pulmonary function parameters, duration of daily walking, the number of steps per day, dyspnea severity and quality of life in normal weight, overweight, and obese patients with stage I and II COPD(26) | - The test results dependent on patient motivation(24,25) - Small sample size(24) - Adherence problem(24,26) - The patients early discharge and stop attendance(24) - No biopsy to assess muscle condition(25) - Self-reports of the measurements(26) | - To elevated the effects of exercise intervention without the medicine use - A follow-up assessment of patients after discharge in order to explore the possible long-term benefits of the intervention - More objective precise measurements are required in the future studies |
| **Diabetes** | - To determine whether interval-based exercise improves postprandial glucose tolerance and free-living glycemia more than oxygen consumption- and time duration-matched continuous exercise(27) - To investigate the effect of short-term toe resistance training on toe pinch force and toe muscle quality(28) | - Small sample size(27,28) - Research design limitation(27) - No comparison group(28) | - The insulin-independent glucose disposal after interval-type exercise - The beneficial effect of greater peak exercise intensity or the cyclic exercise pattern - RCT trial for resistance training |

**References**

1. Weinstein AA, Chin LMK, Keyser RE, Kennedy M, Nathan SD, Woolstenhulme JG, et al. Effect of aerobic exercise training on fatigue and physical activity in patients with pulmonary arterial hypertension. Respir Med [Internet]. 2013;107(5):778–84. Available from: http://dx.doi.org/10.1016/j.rmed.2013.02.006

2. Liu J, Feng W, Zhou J, Huang F, Long L, Wang Y, et al. Effects of sling exercise therapy on balance, mobility, activities of daily living, quality of life and shoulder pain in stroke patients: a randomized controlled trial. Eur J Integr Med [Internet]. 2020;35(February):101077. Available from: https://doi.org/10.1016/j.eujim.2020.101077

3. Kenny M, Gilmartin J, Thompson C. Video-guided exercise after stroke: a feasibility randomised controlled trial. Physiother Theory Pract. 2020;1–12.

4. Mahmood W, Ahmed Burq HSI, Ehsan S, Sagheer B, Mahmood T. Effect of core stabilization exercises in addition to conventional therapy in improving trunk mobility, function, ambulation and quality of life in stroke patients: a randomized controlled trial. BMC Sports Sci Med Rehabil. 2022;14(1):1–9.

5. Arbane G, Douiri A, Hart N, Hopkinson NS, Singh S, Speed C, et al. Effect of postoperative physical training on activity after curative surgery for non-small cell lung cancer: A multicentre randomised controlled trial. Physiotherapy (United Kingdom) [Internet]. 2014;100(2):100–7. Available from: http://dx.doi.org/10.1016/j.physio.2013.12.002

6. Hwang CL, Yu CJ, Shih JY, Yang PC, Wu YT. Effects of exercise training on exercise capacity in patients with non-small cell lung cancer receiving targeted therapy. Supportive Care in Cancer. 2012;20(12):3169–77.

7. Kuehr L, Wiskemann J, Abel U, Ulrich CM, Hummler S, Thomas M. Exercise in patients with non-small cell lung cancer. Med Sci Sports Exerc. 2014;46(4):656–63.

8. Rutkowska A, Jastrzebski D, Rutkowski S, Zebrowska A, Stanula A, Szczegielniak J, et al. Exercise Training in Patients With Non-Small Cell Lung Cancer During In-Hospital Chemotherapy Treatment: A RANDOMIZED CONTROLLED TRIAL. J Cardiopulm Rehabil Prev. 2019;39(2):127–33.

9. Ahn KY, Hur H, Kim DH, Min J, Jeong DH, Chu SH, et al. The effects of inpatient exercise therapy on the length of hospital stay in stages I-III colon cancer patients: Randomized controlled trial. Int J Colorectal Dis. 2013;28(5):643–51.

10. Oechsle K, Aslan Z, Suesse Y, Jensen W, Bokemeyer C, de Wit M. Multimodal exercise training during myeloablative chemotherapy: A prospective randomized pilot trial. Supportive Care in Cancer. 2014;22(1):63–9.

11. Travier N, Velthuis MJ, Steins Bisschop CN, van den Buijs B, Monninkhof EM, Backx F, et al. Effects of an 18-week exercise programme started early during breast cancer treatment: A randomised controlled trial. BMC Med [Internet]. 2015;13(1):1–11. Available from: http://dx.doi.org/10.1186/s12916-015-0362-z

12. Fiuza-Luces C, Padilla JR, Soares-Miranda L, Santana-Sosa E, Quiroga J v., Santos-Lozano A, et al. Exercise Intervention in Pediatric Patients with Solid Tumors: The Physical Activity in Pediatric Cancer Trial. Med Sci Sports Exerc. 2017;49(2):223–30.

13. Morales JS, Padilla JR, Valenzuela PL, Santana-Sosa E, Rincón-Castanedo C, Santos-Lozano A, et al. Inhospital exercise training in children with cancer: Does it work for all? Front Pediatr. 2018;6(December):1–8.

14. Morales JS, Santana-Sosa E, Santos-Lozano A, Baño-Rodrigo A, Valenzuela PL, Rincón-Castanedo C, et al. Inhospital exercise benefits in childhood cancer: A prospective cohort study. Scand J Med Sci Sports. 2020;30(1):126–34.

15. Spreafico F, Barretta F, Murelli M, Chisari M, Gattuso G, Terenziani M, et al. Positive Impact of Organized Physical Exercise on Quality of Life and Fatigue in Children and Adolescents With Cancer. Front Pediatr. 2021;9(June):1–10.

16. Fox L, Cahill F, Burgess C, Peat N, … SRB research, 2017 undefined. Real world evidence: a quantitative and qualitative glance at participant feedback from a free-response survey investigating experiences of a structured exercise. HindawiCom [Internet]. 2017;2017. Available from: https://www.hindawi.com/journals/bmri/2017/3507124/abs/

17. Bryant AL, Walton AML, Pergolotti M, Phillips B, Bailey C, Mayer DK, et al. Perceived benefts and barriers to exercise for recently treated adults with acute leukemia. Oncol Nurs Forum. 2017;44(4):413–20.

18. Platschek A maria, Kehe L, Abeln V, Berthold F, Simon T, Str HK. Computer-Based Exercise Program: Effects of a 12-Week Intervention on Mood and Fatigue in Pediatric Patients With Cancer. Oncology Nursing society. 2015;21(6):280–6.

19. Schram A, Ferreira V, Minnella EM, Awasthi R, Carli F, Scheede-Bergdahl C. In-hospital resistance training to encourage early mobilization for enhanced recovery programs after colorectal cancer surgery: A feasibility study. European Journal of Surgical Oncology [Internet]. 2019;45(9):1592–7. Available from: https://doi.org/10.1016/j.ejso.2019.04.015

20. Dennett AM, Zappa B, Wong R, Ting SB, Williams K, Peiris CL. Bridging the gap: a pre-post feasibility study of embedding exercise therapy into a co-located cancer unit. Supportive Care in Cancer [Internet]. 2021;29(11):6701–11. Available from: https://doi.org/10.1007/s00520-021-06261-2

21. Park JH, Park KD, Kim JH, Kim YS, Kim EY, Ahn HK, et al. Resistance and aerobic exercise intervention during chemotherapy in patients with metastatic cancer: a pilot study in South Korea. Ann Palliat Med. 2021;10(10):10236–43.

22. Mikkelsen MK, Michelsen H, Nielsen DL, Vinther A, Lund CM, Jarden M. ‘Doing What only I Can Do’: Experiences from Participating in a Multimodal Exercise-Based Intervention in Older Patients with Advanced Cancer - A Qualitative Explorative Study. Cancer Nurs. 2022;45(2):E514–23.

23. Kırca K, Kutlutürkan S. The effect of progressive relaxation exercises on treatment-related symptoms and self-efficacy in patients with lung cancer receiving chemotherapy. Complement Ther Clin Pract. 2021;45(September).

24. Borges RC, Carvalho CR. Impact of resistance training in chronic obstructive pulmonary disease patients during periods of acute exacerbation. Arch Phys Med Rehabil [Internet]. 2014;95(9):1638–45. Available from: http://dx.doi.org/10.1016/j.apmr.2014.05.007

25. Torres-Sánchez I, Valenza MC, Cabrera-Martos I, López-Torres I, Benítez-Feliponi Á, Conde-Valero A. Effects of an Exercise Intervention in Frail Older Patients with Chronic Obstructive Pulmonary Disease Hospitalized due to an Exacerbation: A Randomized Controlled Trial. COPD: Journal of Chronic Obstructive Pulmonary Disease. 2017;14(1):37–42.

26. Yilmaz FT, Aydin HT. The effect of a regular walking program on dyspnoea severity and quality of life in normal weight, overweight, and obese patients with chronic obstructive pulmonary disease. Int J Nurs Pract. 2018;24(3):1–11.

27. Karstoft K, Christensen CS, Pedersen BK, Solomon TPJ. The acute effects of interval-Vs continuous-walking exercise on glycemic control in subjects with type 2 diabetes: A crossover, controlled study. Journal of Clinical Endocrinology and Metabolism. 2014;99(9):3334–42.

28. Kataoka H, Miyatake N, Kitayama N, Murao S, Tanaka S. A pilot study of short-term toe resistance training in patients with type 2 diabetes mellitus. Diabetol Int. 2017;8(4):392–6.
